# Synthesis and Structural Investigation of New Bio-Relevant Complexes of Lanthanides with 5-Hydroxyflavone: DNA Binding and Protein Interaction Studies

**DOI:** 10.3390/molecules21121737

**Published:** 2016-12-16

**Authors:** Alexandra-Cristina Munteanu, Mihaela Badea, Rodica Olar, Luigi Silvestro, Constanţa Dulea, Constantin-Daniel Negut, Valentina Uivarosi

**Affiliations:** 1 Department of General and Inorganic Chemistry, Faculty of Pharmacy, “Carol Davila” University of Medicine and Pharmacy, 6 Traian Vuia Str., 020956 Bucharest, Romania; alexandra.ticea@umf.ro; 2 Department of Inorganic Chemistry, Faculty of Chemistry, University of Bucharest, 90-92 Panduri Str., 050663 Bucharest, Romania; e_m_badea@yahoo.com (M.B.); rodica_m_olar@yahoo.com (R.O.); 3 PharmaServ. International SRL, 52 Sabinelor Str., 050853 Bucharest, Romania; dreispharmasl@aol.com (L.S.); dulea_c@yahoo.com (C.D.); 4 Horia Hulubei National Institute of Physics and Nuclear Engineering (IFIN-HH), IRASM Radiation Processing Department, Reactorului Str. 30, 077125 Magurele-Ilfov, Romania; dnegut@nipne.ro

**Keywords:** lanthanides, chelates, density functional calculations, calf thymus DNA, HSA, transferrin

## Abstract

In the present work, we attempted to develop new metal coordination complexes of the natural flavonoid 5-hydroxyflavone with Sm(III), Eu(III), Gd(III), Tb(III). The resultant hydroxo complexes have been characterized by a variety of spectroscopic techniques, including fluorescence, FT-IR, UV-Vis, EPR and mass spectral studies. The general chemical formula of the complexes is [Ln(C_15_H_9_O_3_)_3_(OH)_2_(H_2_O)_x_]·*n*H_2_O, where Ln is the lanthanide cation and x = 0 for Sm(III), x = 1 for Eu(III), Gd(III), Tb(III) and *n* = 0 for Sm(III), Gd(III), Tb(III), *n* = 1 for Eu(III), respectively. The proposed structures of the complexes were optimized by DFT calculations. Theoretical calculations and experimental determinations sustain the proposed structures of the hydroxo complexes, with two molecules of 5-hydroxyflavone acting as monoanionic bidentate chelate ligands. The interaction of the complexes with calf thymus DNA has been explored by fluorescence titration and UV-Vis absorption binding studies, and revealed that the synthesized complexes interact with DNA with binding constants (K_b_) ~ 10^4^. Human serum albumin (HSA) and transferrin (Tf) binding studies have also been performed by fluorescence titration techniques (fluorescence quenching studies, synchronous fluorescence spectra). The apparent association constants (K_a_) and thermodynamic parameters have been calculated from the fluorescence quenching experiment at 299 K, 308 K, and 318 K. The quenching curves indicate that the complexes bind to HSA with smaller affinity than the ligand, but to Tf with higher binding affinities than the ligand.

## 1. Introduction

Flavonoids are among the most intensively studied and diverse class of plant metabolites. Animals and humans do not produce this group of phytochemicals, but consume them daily through food. Flavonoids can influence various cellular processes, including membrane transport, protein assembly, cell signal transduction, intracellular reduction-oxidation processes, and gene expression [1].

Due to the favorable spatial arrangement of the hydroxyketone moiety in their structures, flavonoids have been successfully used in coordination chemistry as ligands. Therefore, flavonoids have the ability to bind metal ions in multiple oxidation states, forming stable coordination complexes [2]. The metal complexes thus formed show in many cases greater therapeutic potential than the parent ligand.

Lanthanide (Ln) coordination chemistry has registered substantial progress in the last two decades due to its numerous applications. Trivalent lanthanide cations behave as ‘‘hard’’ Lewis acids. Therefore, their affinities toward ‘‘hard’’ ligands with oxygen donor atoms, such as flavonoid derivatives, will be very strong [3]. The design of Ln complexes is focused on potential application in areas such as extraction and separation, catalysis, nonlinear optics (NLO), luminescent probes, magnetism, or is pointing towards therapeutic potential as antioxidant, anticancer [4,5,6], or antimicrobial agents [7]. Because of their long wavelength emission and lifetimes, Ln complexes have the potential for application as sensors and probes in biological and biomedical research [8].

Studies of the interaction between some lanthanide ions and flavonoid molecules led to the isolation and characterization of solid complexes derived from chrysin, Ln(C_15_H_9_O_4_)_3_·4H_2_O (where: C_15_H_9_O_4_ is deprotonated chrysin) [9], [Ln(chrysin)_3_] [10], [LnL_2_(H_2_O)_2_Cl] (L: chrysin) [11], quercetin, [Ln(quercetin)_3_]·*n*H_2_O [12], or morin, Ln(morin)_3_ [13], Ln(C_15_H_9_O_7_)_3_·*n*H_2_O, where *n* = 6 or 8, C_15_H_9_O_7_ = morin [14,15].

Interactions of flavonoid metal complexes with DNA are well documented in literature, as they are relevant for the rational design of efficient antitumor and antimicrobial agents [16] or potential probes of DNA structure and conformation [17]. Ln-flavonoids complexes interact with DNA in a non-covalent way, mostly by intercalation between DNA base pairs or outside bonding. Intermolecular H-bonds and electrostatic forces may also be involved in the binding process [17,18,19,20,21]. The values of the binding constants vary between 10^2^–10^7^ M^−1^.

**Figure 1:**
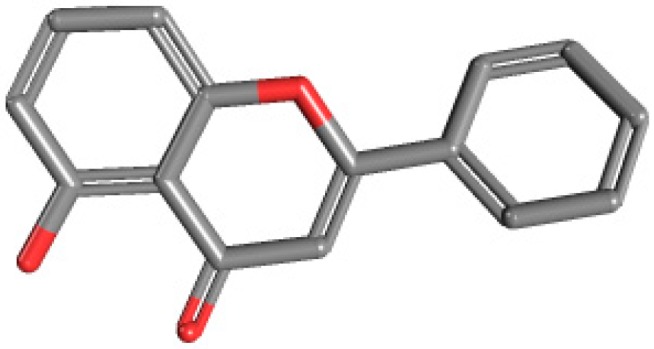
Primuletin (5-hydroxyflavone).

Human serum albumin (HSA) is the most abundant extracellular protein of the circulatory system. Studies of drug-HSA interactions may bring valuable information regarding the pharmacokinetics of that substance, thus being useful for drug design and screening [22]. Transferrin (Tf) is involved in metal ions transportation throughout the organism (mainly iron) [23]. Cancer cells, as active and rapidly proliferating cells, express high levels of transferrin receptors. Consequently, transferrin is a potential drug carrier for targeted delivery into tumor cells [24]. To our knowledge, there are no reported studies on the interaction between Ln-flavonoid complexes and HSA or Tf. Primuletin (5-hydroxyflavone; 5-hydroxy-2-phenyl-4*H*-1-benzopyran-4-one, Figure 1) occurs naturally in *Primula* sp. [25] and *Dionisya* sp. [26]. In comparison to other flavonoids, the biological activity of 5-hydroxyflavone (5-HOF) is yet poorly explored. To date, it has been reported that 5-HOF acts as an agonist of calcium-activated and ATP-sensitive potassium channels [27], and has strong vasorelaxing and androgen receptor antagonistic activity [28].

Due to the presence of a chelating 5-hydroxy-4-keto moiety, 5-hydroxyflavone can act as a bidentate ligand toward ions of *p*-, *d*-, and *f*-metals. Several metal complexes of 5-hydroxyflavone have been obtained in solution or synthesized in solid state [29,30,31,32,33,34]. However, their applications are limited: 5-hydroxyflavone-Al(III) complex has been designed as a fluorescent fluoride ion probe [33] and [Be(5-HOF)_2_] has been tested as electroluminescent material in organic light-emitting diodes [34].

Our study aims to present the synthesis and structural characterization of four new solid complexes of 5-hydroxyflavone with some lanthanide ions: samarium(III), europium(III), gadolinium(III), and terbium(III). Studies regarding DNA- and protein-interactions have been performed in order to assess the potential biological applications of the complexes.

## 2. Results and Discussion

### 2.1. Synthesis

**Scheme 1:**
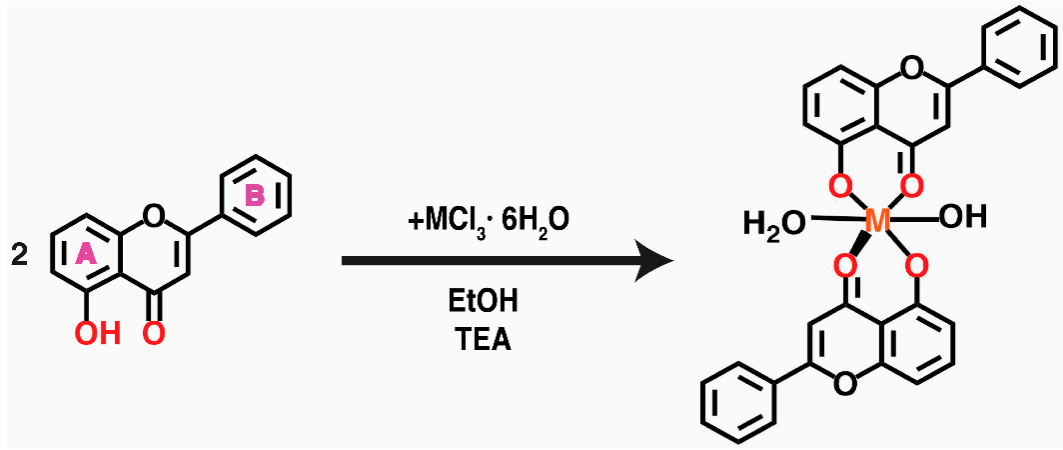
The reaction scheme for the synthesis of complexes, where MCl_3_ = the lanthanide salt, EtOH = ethanol, TEA = triethylamine.

The complexes reported in this paper were obtained according to the general synthesis scheme depicted in Scheme 1.

The abbreviated formulas that we have attributed to the four complexes and that will be used in this work are as follows:

[Sm(OH)L_2_] (**1**)

[Eu(OH)L_2_(H_2_O)]·H_2_O (**2**)

[Gd(OH)L_2_(H_2_O)] (**3**)

[Tb(OH)L_2_(H_2_O)] (**4**)

where

- L = C_15_H_9_O_3_, deprotonated 5-hydroxyflavone
- The metal complexes were assigned the following codes, that will be used further in this article: (**1**), (**2**), (**3**), (**4**); 5-hydroxyflavone will be used as 5-HOF

Adding triethylamine assures the deprotonation of the ligand, so that 5-hydroxyflavone acts as a monoanionic bidentate chelate coordinated via 5-hydroxy-4-keto moiety.

### 2.2. Physicochemical Characterization

The colour of the prepared complexes is yellow. Complexes (**1**)–(**4**) have been found to be very soluble in DMF and DMSO and moderately soluble in chloroform. However, complex (**1**) is less soluble in DMSO than the other three complexes. Their solubility in ethanol, methanol, acetonitrile, acetone and water is very low. Low molar conductance values show that the complexes are non-electrolytes in DMSO.

#### 2.2.1. IR Spectra

The spectra of the ligand and the complexes provide information about the coordination of Sm(III), Eu(III), Gd(III), and Tb(III) ions to 5-hydroxyflavone. The intense broad band between 2600 and 3200 cm^−1^ which appears in the IR spectra of 5-hydroxyflavone [35] is due to the the strong intramolecular hydrogen bond involving the phenolic hydroxyl in the free ligand. A sharp band at 3059 cm^−1^ corresponds to the stretching vibration ν(C-H). In the IR spectra of the complexes (except for (**1**)) a broad band between 2600 and 3600 cm^−1^ is found, assigned to the presence of water of hydration in their structure. The intense absorption bands due to ν(C=O) in the free ligand, placed at 1654 and 1615 cm^−1^ appear shifted to lower frequencies in the spectra of the complexes. The displacement of ~20 cm^−1^ can be explained by the involvement of the carbonyl oxygen in metal binding. The strong band which appears at 1587 cm^−1^ in the IR spectrum of the ligand was assigned to ν(C=C). In the IR spectra of the complexes, this band is slightly shifted, indicating that this bond is not involved in coordination. In the IR spectrum of the ligand a strong band appears at 1298 cm^−1^, assigned to the coupled vibration ν(C-O) + δ(OH). The same band appears very weak in the spectra of the complexes, which indicates that 5-hydroxyflavone is coordinated in its deprotonated form. The ν(C-O-C) frequency does not appear shifted in the spectra of the complexes, which confirms that the ring oxygen is not involved in coordination. Moreover, in the spectra of the complexes, the presence of ν(M-O) stretching vibration at ~560 cm^−1^ indicates metal binding, since the spectrum of the ligand does not exhibit this band [35,36]. 

#### 2.2.2. UV-Vis-NIR Spectra

**Figure 2:**
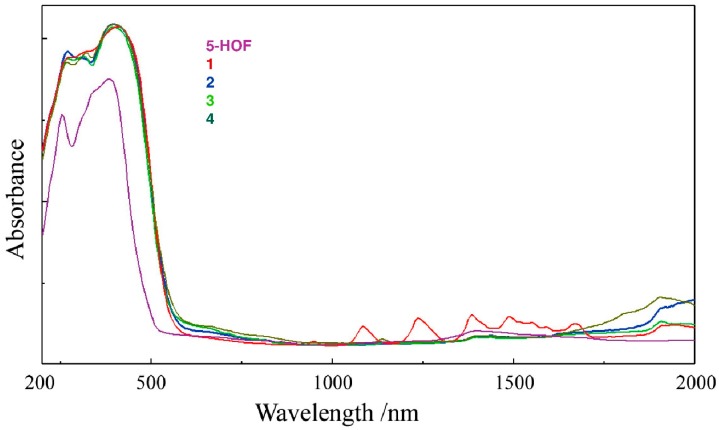
Electronic spectra of the ligand and the complexes (recorded on solid probes).

The ligand, 5-hydroxyflavone, exhibited two absorption maxima originated from π-π* transitions at 385 nm (band I) and 255 nm (band II). Band I is related to the transition localized within the B ring (cinnamoyl system), whereas band II is due to the transitions in the A ring (benzoyl system) [37,38] (the A and B ring are depicted in Scheme 1). Bathochromic shifts regarding these two bands are observed in the spectra of the complexes, which can be related to the extension of the conjugated system of the ligand with coordination [39]. The shoulder that appears at 340 nm in the electronic spectrum of the ligand, is also present in the electronic spectra of complexes as shoulder or maximum, with a hypsochromic shift (Δλ~20–25 nm). UV-Vis data for ligand and complexes are presented in Table S1. In the NIR region of spectrum, the characteristic f-f transitions form the ground state ^6^H_5/2_ to ^6^F multiplet of Sm^3+^ ion (4f^5^) are observed (Figure 2) [40].

#### 2.2.3. Mass Spectra

The compounds analysed gave intense pseudomolecular ions by protonation as well as adducts with one or two water molecules ions (Table S2 columns 2nd, 3rd, 4th); for all products the spectra were in agreement with the natural isotopic abundances of the lanthanides involved. Protonated pseudomolecular ions of all compounds, with the exception of that with Sm(III) formed, by collision with nitrogen, intense fragment ions corresponding to the loss of a ligand molecule (Table S2, 5th column). All data obtained by mass spectrometry confirm that these compounds have a 1:2 molar ratio metal ion: ligand (Figure S1).

#### 2.2.4. Thermal Behavior

Thermal analysis is frequently used in order to obtain useful information concerning both the composition and stability of the complexes. As a result, the thermal behaviour of the four new lanthanide complexes was investigated in air by simultaneous TG/DTA analysis. The thermal decomposition data are summarized in Table 1 and will be discussed as follows.

The TG, DTG and DTA curves registered for Sm(III) complex (**1**) are shown in Figure S2 and indicate that this compound is anhydrous and stable up to 320 °C and then undergoes oxidative degradation of the organic part.

For the other three complexes, (**2**)–(**4**), the thermal decomposition curves are quite similar, consisting in water molecules elimination and oxidative degradation of organic ligands. For the europium complex which possesses both coordination and crystallization water molecules the thermal decomposition starts at 170 °C, this being the lowest decomposition starting temperature. All anhydrous species are stable up to 320 °C.

In the final step the organic part oxidative degradation occurs, accompanied by several overlapped exothermic processes as can be noticed on DTA curve. All these processes lead to the most stable species M_2_O_3_ as final products. 

In conclusion, according to DTG and DTA curves, the thermal transformations are complex processes consisting in water (crystallization or coordination) elimination, thermolysis and oxidative degradation of 5-hydroxyflavone (Figure S2).

#### 2.2.5. Fluorescent Properties

The fluorescence emission spectra (Figures S3 and S4) were recorded in chloroform at a concentration of 5 µg/mL at the excitation wavelengths of 360 nm and 440, respectively, both for ligand and complexes. The results let us draw the following conclusions:

(i) 5-hydroxyflavone does not exhibit fluorescence in the stated conditions;
(ii) at the excitation wavelength of 360 nm, two new bands appeared at 411 and 436 nm in the fluorescence emission spectra of the complexes (**1**) and (**2**).
(iii) At the excitation wavelength of 440 nm, a new band appeared at 481 nm in the fluorescence emission spectra of complex **1**.

The new bands that appeared in the emission spectra of complexes could be appreciated as evidence for the formation of a chelate ring via coordination of the metal ion, which increases the rigidity of the ligand structure and enhances the fluorescence quantum yield by reducing the probability of non-radiative dissipation process.

#### 2.2.6. EPR Spectroscopy

Gd^3+^ ions, with a 4f^7^ electronic configuration, have a larger relaxation time (10^−9^–10^−10^ s) [41], exhibiting detectable EPR signals at room temperature. Tb(III) and Eu(III) ions, due to their short relaxation times (10^−13^ s), do not normally show EPR signals at room temperature [42]. 

**Figure 3:**
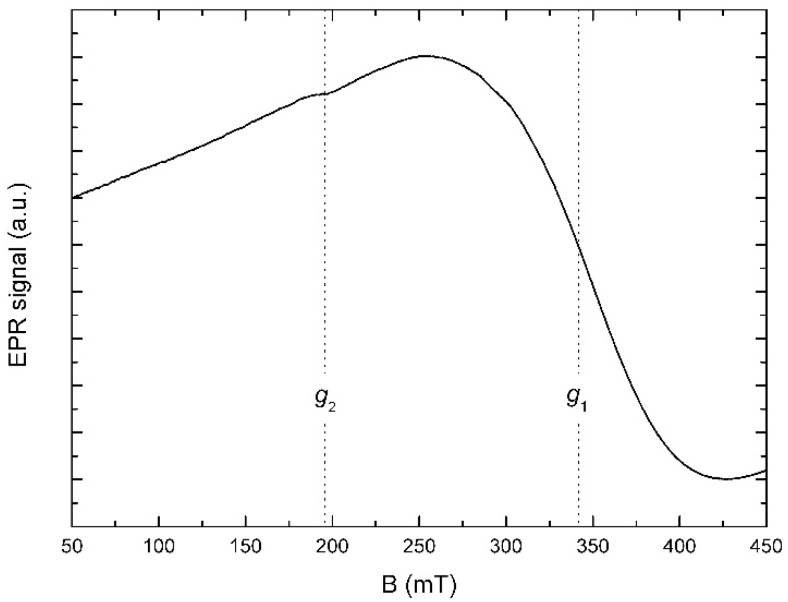
EPR spectrum of **3** (powder) recorded at room temperature.

Figure 3 shows the solid state EPR spectrum of **3**, recorded at room temperature. The spectrum is dominated by a very broad, slightly asymmetrical line (marked by *g*_1_ in the figure) with an effective *g* factor of 1.99 and the peak-to-peak linewidth (Δ*B*_pp_) of about 175.5 mT. Additionally, a weak, unresolved resonance (*g*_2_) can be observed at *g* = 3.47, with a Δ*B*_pp_ ≈ 29.17 mT. The resonance at *g* ~ 2.0 is typical for Gd(III) located in low-symmetry sites [43]. The paucity of fine structure and line broadening indicate a week crystal field [44].

### 2.3. DFT Calculations

**Figure 4:**
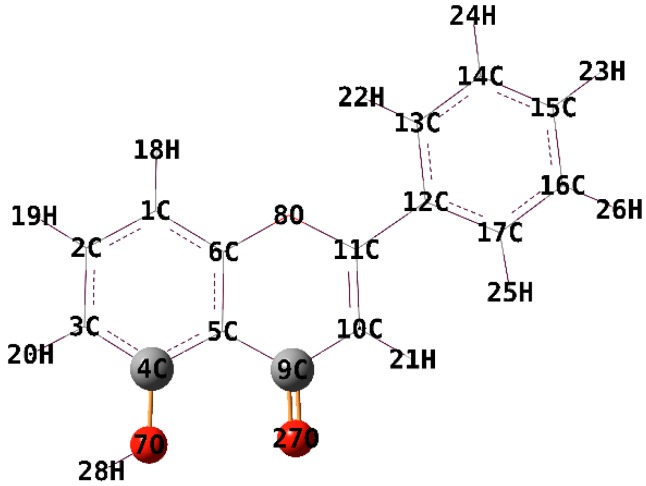
The optimised structure of the electronic ground state of 5-hydroxyflavone at the B3LYP level of theory (6-31G(d) basis).

**Figure 5:**
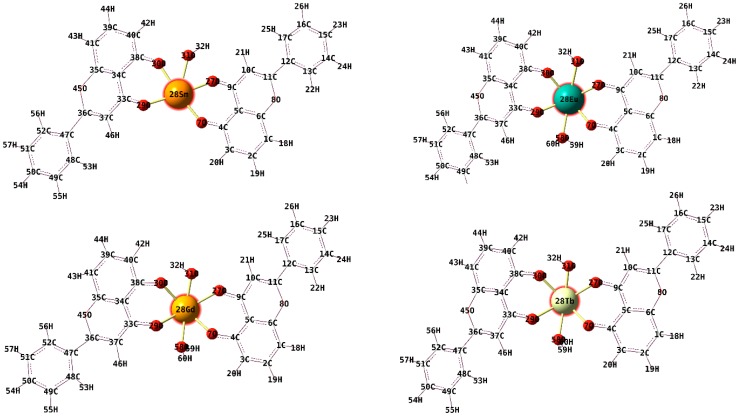
The optimised structure of the electronic ground state of complexes (**1**)–(**4**) at the uB3LYP level of theory (6-31G(d) basis set for C, H atoms; 6-31G+(d,p) for O atoms; MWB28 for lanthanide atoms).

The fully optimized geometries of the ligand and complexes **1**–**4** and the symbols and the labels of atoms are shown in Figure 4 and Figure 5. 

The B3LYP functional is used frequently and provides good results. We have used the unrestricted version, uB3LYP. The optimizations were employed under no symmetry restrictions and vibrational frequencies were additionally calculated at the same level of theory in order to confirm that the optimized structures are true minima (with no imaginary frequencies). 

Selected bond lengths, angles, charge density, total energy and total dipole moment are presented in Table 2 and Table 3. 

The coordination sphere around the metal centre in complex (**1**) (Figure 4) is made up of the following atoms: O7, O27 and O29, O30 (belonging to 5-hydroxyflavone) and O31 (belonging to the hydroxyl group). The coordination sphere around the metal centre in complexes (**2**)–(**4**) has one extra O atom, belonging to a water molecule. 

In complexes (**2**)–(**4**), the O-M-O bond angles in the complexes vary between 46°–106° which indicate a distorted octahedral geometry. The values of the dihedral angles around the lanthanide ion in complexes (**2**)–(**4**) (Figure 5), are far from 0° or 180° which indicate that the lanthanide ion and the donating sites do not lie in the same plane. In complex (**1**), the corresponding dihedral angles are close to 0° (−1.366°) and 180° (179.599°), indicating that Sm^3+^ is situated in the same plane with the moieties involved in coordination [45].

### 2.4. In Vitro Interactions with Biological Macromolecules

#### 2.4.1. DNA Binding Studies

Drugs bind to DNA both covalently (irreversibly) and non-covalently (reversibly). Covalent binding to DNA often leads to complete inhibition of vital processes and subsequent cell death. Reversible binding with double-stranded DNA mainly follows three patterns: the drug molecules interact: (*i*) with the external negatively charged nucleic sugar-phosphate structure, via electrostatic binding; (*ii*) with the minor and/or major grooves of DNA double helix-usually implies electrostatic forces, van der Waals interactions, hydrogen bonds, dative bonds and/or hydrophobic interactions; or (*iii*) the drug molecules intercalate between DNA base pairs (the DNA-drug complex is stabilized by the π-π* stacking interactions between the aromatic systems of the tested compound and DNA bases). Intercalators are usually planar heterocyclic compounds which stack between adjacent base pairs in the DNA structure. Flavonoids and their metal complexes possess such planar structures due to the conjugated π-bond ring system, and, therefore, have the potential to intercalate between DNA bases and to form stable complexes. Several studies revealed that the above mentioned compounds bind to DNA via groove interactions or intercalation [46,47].

##### UV-Vis Spectroscopy Studies

In order to explore the mechanism of DNA-Ln(III) complexes interaction, the spectrophotometric titration of the complexes in the presence of highly polymerized CT-DNA was performed. We evaluated the changes in the recorded spectra, like hypsochromic (blue-shift), bathochromic (red-shift) or hypochromic effects, commonly considered as evidence of DNA-drug interactions [48]. 

Binding in a nonintercalative manner with DNA, generally results in hyperchromism due to the strong disturbances induced in the DNA double helical structure. Nonetheless, intercalation generally results in hypochromism and red shift due to strong π-π* stacking interactions between an aromatic moiety of the ligand and DNA nucleotides.

**Figure 6:**
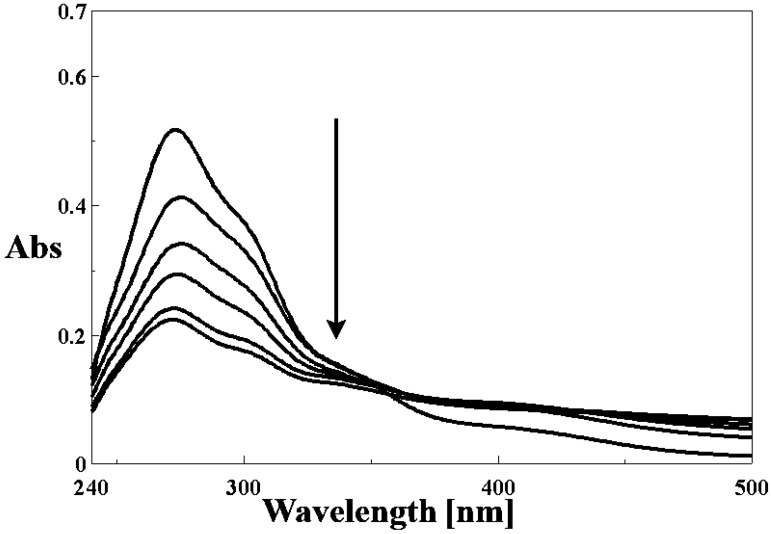
Absorption spectra of complex **2** in the absence and presence of increasing amounts of DNA, respectively; [complex] = 15 μM; [DNA] = 0; 5; 10; 15; 25; 30 μM. The arrow shows the absorption changes on increasing DNA concentration.

In the UV spectra of the three complexes after the addition of increasing DNA concentrations, the band centered at ~273 nm exhibits a significant hypochromism of ~40% (Figure 6 and Figure S5), suggesting strong binding to CT DNA, possibly by intercalation [49]. Furthermore, in regard to spectral general features, a strong resemblance of the four compounds can be noticed, therefore similar binding modes to CT-DNA are expected. Complex (**1**) was also tested in the same conditions, but the results did not follow any recognizable pattern of interaction with CT-DNA, if any.

**Figure 7:**
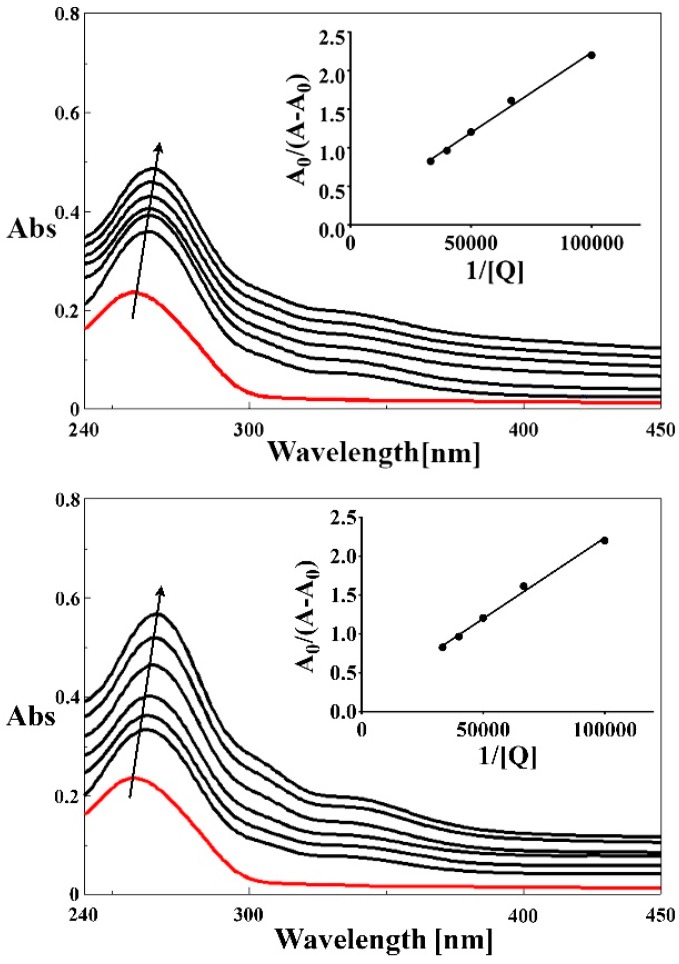
Absorption spectra of DNA in the absence and presence of increasing amounts of 5-HOF and complex (**2**). [DNA] = 15 μM; [compound] = 0; 5; 10; 15; 20; 25; 30 μM. Insert figures represent the plots of A_0_/(A − A_0_) vs. 1/[Q]. The arrows show the absorption changes on increasing concentration of the tested compound.

The following equation has been used to calculate the binding constant of 5-hydroxyflavone and its Ln(III) complexes [22]:

$$\frac{A_{0}}{A-A_{0}}=\frac{\varepsilon_{c}}{\varepsilon_{\mathrm{DNA}-c}-\;\varepsilon_{\;c}}-\frac{\varepsilon_{c}}{\varepsilon_{\mathrm{DNA}-c}-\;\varepsilon_{\;c}}\times \frac{1}{K\left[Q\right]},$$

where A_0_ and A represent the absorbance of DNA in the absence and presence of the ligand and the complexes (**2**), (**3**), (**4**) at λ_max_ = 258 nm, ɛ*_c_* and ɛ_DNA−c_ are the absorption coefficients of the tested compounds and compound-DNA system, respectively. The plot of A_0_/(A − A_0_) vs. 1/[Q] is constructed by using the absorption titration data and linear fitting (Figure 7 and Figure S6). The calculated binding constants for 5-HOF and complexes (**2**), (**3**), (**4**), respectively are presented in Table 4.

Moreover, as comparing to ethidium bromide (EB), which is a classical intercalator, with a K_b_ ~ 10^5^ M^−1^, these values are not much smaller. Interaction of the four compounds with CT-DNA is weaker than the DNA interaction with typical intercalators. However, an electrostatic and/or intercalative interaction may explain EB displacement from the EB-DNA complex.

##### Competitive Binding Studies with Ethidium Bromide Using Fluorescence Spectroscopy

Ethidium bromide (3,8-diamino-5-ethyl-6-phenylphenanthridinium bromide, EB) is a fluorescent dye, which has a planar phenanthridinium ring in its structure. Due to the intercalation of this planar ring between adjacent base pairs on the DNA double helix, EB emits intense fluorescence in the presence of CT DNA, after the formation of an EB-DNA complex. Therefore, an EB-bound CT-DNA solution (2 µM EB + 10 µM DNA) has been used as a spectral probe. 

When the metal complexes bind to DNA, a decrease in the emission intensity of the EB-DNA complex is recorded (a quenching effect). The variation of the fluorescence emission intensity gives some information about the DNA binding affinity of the metal complexes and usually indicates the existence of stacking interactions (intercalation) between the DNA base pairs.

Data from the competitive binding studies have been plotted according to the classical Stern–Volmer equation [50]:

I_0_/I = 1 + K_sv_∙[Q],

where I_0_ and I represent the fluorescence intensities in the absence and presence of the compound respectively, and [Q] is the concentration of the tested compound. 

**Figure 8:**
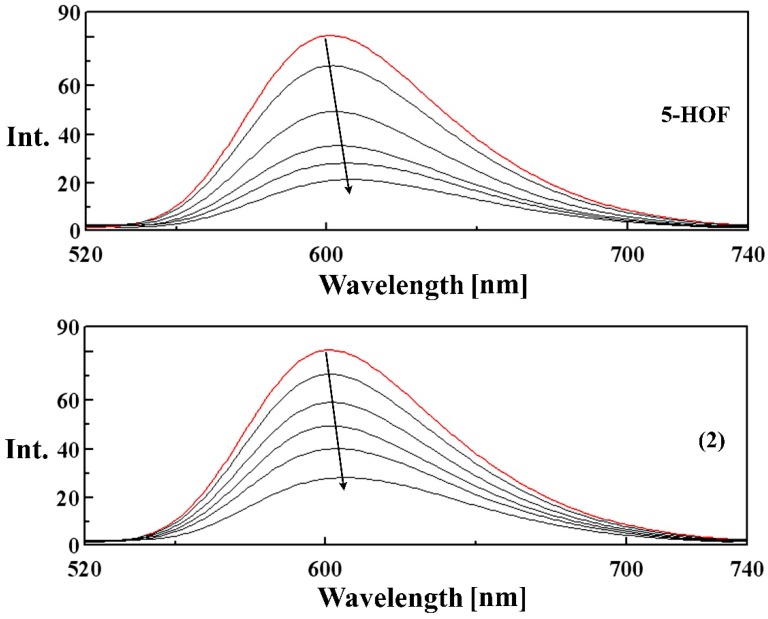
Fluorescence spectra of the binding of ethidium bromide (EB) to DNA in the absence (red line) and in the presence (black lines) of increasing amounts of the ligand and complex (**2**) λex = 500 nm, [EB] = 2 μM, [DNA] = 10 μM, [compound] = 10, 15, 20, 25, 30 μM. Arrows indicate the changes in fluorescence intensities upon increasing the amounts of the tested compound.

The Ksv value is calculated as the slope of I_0_/I versus [Q] linear regression plot (Table 5). The emission spectra of EB-DNA complex in the absence and presence of increasing concentrations of the ligand and complexes (**2**)–(**4**) are shown in Figure 8 and Figure S7. The quenching curves indicate that the complexes bind to CT-DNA with higher binding affinities than the ligand, in the order: 5-HOF < (**4**) < (**2**) < (**3**).

A small red shift (~5 nm) appears in the fluorescence spectra of EB-DNA upon binding to the tested compounds, due to the induced changes in the bond lengths and angles in the structure of the ligand (Figure 8).

#### 2.4.2. HSA and Tf Binding Studies

Insights into the nature of binding can be obtained from fluorescence quenching studies by monitoring the fluorescence intensities. Fluorescence spectroscopy is an effective method to explore the interactions of small molecules with proteins, such as HSA and Tf, which contain tryptophan residues. Variations of the fluorescence intensity of the chromophore may reflect changes in the microenvironment surrounding the fluorophore. 

##### Fluorescence Quenching Mechanism

HSA exhibits a strong fluorescence emission peak at ~340 nm (when λ_exc_ = 280 nm) owing to the Tryptophan (Trp)-214 residue. The fluorescence intensity of Trp-214 may change when HSA interacts with other molecules. The quenching effect of 5-HOF and compounds (**1**)–(**4**) on the fluorescence intensity of HSA at 299, 308, 318 K are shown in Figures S8–S10, respectively. Trp, tyrosine (Tyr) and phenylalanine (Phe) amino acid residues in Tf have intrinsic fluorescence depending on the excitation wavelength. However, with fluorescence excitation at 295 nm, the tryptophan emission fluorescence is highly dominant [51]. The quenching effect of 5-HOF and compounds (**1**)–(**4**) on the fluorescence intensity of Tf at 299, 308, 318 K are shown in Figures S11–S13, respectively.

The intensity of the broad emission band decreased upon addition of increasing concentrations of the five tested compounds; the maximum emission wavelength of HSA exhibits a hypsochromic shift of up to 9 nm. This blue-shift effect indicates that the microenvironment around the tryptophan residue was disturbed, that the Trp residue is located in a more hydrophobic environment, becoming less exposed to the solvent [52]. On the other hand, the maximum emission wavelength of Tf does not appear shifted.

A modified Stern–Volmer equation was applied to the HSA and Tf fluorescence quenching data [23]:

$$\frac{F_{0}}{F_{0}-F}=\frac{1}{f_{a}K_{\mathrm{sv}}\left[Q\right]}+\frac{1}{f_{a}}$$

where F_0_ and F are the relative fluorescence intensities of HSA and Tf, respectively, in the absence and presence of our tested compounds, respectively, f_a_ is the fraction of fluorophore accessible to the quencher, [Q] is the concentration of the quencher, and K_SV_ is the Stern–Volmer quenching constant. The resulted curves, depicted in Figures S14 and S15 show good linear relationships. Therefore, parameters K_a_ and f_a_ were calculated from the slope and intercept, respectively, in the plot of F_0_/(F_0_ − F) vs. 1/[Q].

K_sv_ = K_q_τ_0_,

where K_q_ is the bimolecular quenching rate constant and τ_0_ is the lifetime of the fluorophore in the absence of the quencher.

The Stern–Volmer quenching constant (K_SV_, M^−1^) and the quenching rate constant (K_q_, M^−1^ s^−1^) for the ligand and the complexes which resulted from our studies are given in Table S3 (HSA) and Table S4 (Tf). These values suggest good binding affinity of the complexes with HSA and Tf. K_q_ values (>10^11^ M^−1^ s^−1^) indicate the existence of a static quenching mechanism [53]. 

The following equation was used to determine K_a_ and n, where n is the number of binding sites [54]:

$$lg\frac{F_{0}-F}{F}=lgK_{A}+nlg[Q]$$

Plots of lg[(F_0_ − F)/F] vs. lg[Q] for HSA and Tf-tested compounds systems 5-HOF, (**1**)–(**4**)) are represented in Figures S16 and S17. Temperature did not vary in a broad range, therefore the value of the enthalpy change could be considered to be constant and the van’t Hoff equation can be used in order to estimate the thermodynamic parameters:

lnK = −ΔH/RT + ΔS/R

where K is the binding constant at the corresponding temperature (K), ΔH is the enthalpy change, R is the gas constant and ΔS is the entropy change. The values of ΔH and ΔS were obtained from the slope and the y intercept of a lnK vs. 1/T. Moreover, the free energy change (ΔG) can be calculated from the following relationship:

ΔG = ΔH − TΔS

The negative values of ΔG presented in Tables S3 and S4 indicate that the HSA and Tf binding processes are spontaneous. Moreover, since both ΔH and ΔS values are negative for all the tested compounds, hydrogen bonds or van der Waals forces most probably play important roles in the compound-proteins interactions [55].

##### Changes of Tf Conformation Induced by the Interaction with the Tested Compounds 

Conformational changes of a protein in terms of secondary or tertiary structures can be altered upon binding to small molecules. Synchronous fluorescence spectra bring information about the molecular surroundings of proteins. One major advantage of this method compared to conventional emission spectra is that even small variations in the absorption and/or emission bands affect the corresponding band in a synchronous spectrum much more than in the case of a conventional emission spectrum. This improves spectral resolution, thus facilitating the analysis of minor structural perturbations. Therefore, we have used synchronous scan fluorescence spectroscopy in order to verify the change of the structure of Tf when binding to our compounds, in the presence of increasing concentrations of our complexes and the ligand [55,56,57].

**Figure 9:**
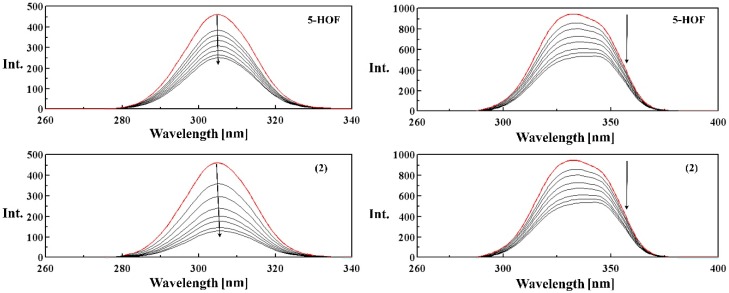
Fluorescence synchronous spectra of 5-HOF and complex (**2**) at Δλ = 15 nm (**left**) and 60 nm (**right**). [Tf] = 10 µM, pH = 7.4; [tested compound] = 0; 5; 7.5; 10; 12.5; 15; 17.5; 20 µM. Arrows indicate the changes in fluorescence intensities upon increasing the amounts of the tested compound.

Synchronous fluorescence spectra offer the characteristics of the tyrosine and tryptophan residues of transferrin when the wavelength interval (Δλ) between excitation and emission is 15 nm or 60 nm. Figure 9 and Figure S18 show the synchronous spectra of Tf in the absence and presence of different concentrations of our tested compounds. We have noticed that the emission maximum of tyrosine does not shift, whereas there was a red shift (up to 12 nm) of tryptophan residues fluorescence upon addition of the tested compounds. This indicates that the conformation of Tf around the tryptophan residues had changed, the polarity around this region had increased and the hydrophobicity had decreased [56]. 

## 3. Materials and Methods

### 3.1. Materials

All reagents and solvents were of analytical reagent grade and were used without further purification. 5-hydroxyflavone, SmCl_3_·6H_2_O, EuCl_3_·6H_2_O, GdCl_3_·6H_2_O and TbCl_3_·6H_2_O, human transferrin and human serum albumin and double-stranded calf-thymus DNA were purchased from Sigma Aldrich Chemical Co. (Schnelldorf, Germany). All other chemicals and solvents were commercially available, and used without further purification.

### 3.2. Analytical Methods

Elemental analyses (for C, H, N, S) were performed using a PE 2400 analyser (Perkin Elmer, Billerica, MA, USA). The conductivity was measured with a Consort C830 (Turnhout, Belgium) conductimeter with an SK10T platinum electrode embedded in glass (cell constant 1.0 cm^−1^). IR spectra were recorded using KBr pellets on a FT-IR VERTEX 70 spectrometer (Bruker, Billerica, MA, USA) in the range 400–4000 cm^−1^. Electronic spectra by the diffuse reflectance technique, with spectralon as reference sample, were recorded in the range 200–2000 nm, on a V 670 spectrophotometer (Jasco, Tokyo, Japan). Fluorescence spectra were recorded on a Jasco FP 6500 spectrofluorometer. 

Mass spectra were recorded, in positive mode scanning over a range *m*/*z* 100–1000, with an API 5000 (SCIEX-Canada, Concord, ON, Canada) triple quadrupole mass spectrometer equipped with a pneumatically assisted electrospray (ESI) source (Turbo spray model from SCIEX). Samples were dissolved in methanol at 10 µg·mL^−1^ and infused in the ESI source at 7 µL·min^−1^. Collision spectra (MS/MS), from protonated pseudomolecular ions, were acquired on the same instrument employing nitrogen as collision gas.

The heating curves (TG, DTG and DTA) were recorded using a Labsys 1200 SETARAM instrument (Setaram, Caluire, France) with a sample weight between 10 and 14 mg over the temperature range of 30–1000 °C and a heating rate of 10 °C·min^−1^. The measurements were carried out in synthetic air atmosphere (flow rate 17 mL·min^−1^), using alumina crucible. Sample masses were weighed on a microbalance with a resolution of 0.1 mg. 

EPR measurements were carried out by means of a continuous-wave X-band spectrometer (MiniScope MS200, Magnettech, Berlin, Germany) equipped with a H102 rectangular resonator and 100 kHz modulation frequency. The spectrum was recorded at room temperature (24–26 °C) using a microwave power of 10 mW with modulation field amplitude of 0.2 mT.

### 3.3. Synthesis and Characterization of Complexes

Synthesis of complexes was carried out using the following general procedure: in a round bottomed flask provided with an electromagnetic stirrer, an ethanolic solution (25 mL) of 5-hydroxyflavone (1 mmol, 0.238 g), deprotonated with triethylamine (1 mmol, 140 µL) was added to an ethanolic solution (5 mL) of lanthanide chlorides (0.5 mmol). The reaction mixture was refluxed for 3 h. The yellow products formed were filtered off, washed several times with small amounts of a hot mixture of ethanol:water (1:1, *v*/*v*), and dried in an exsicator. The reaction scheme is shown in Scheme 1. 

Complex (**1**)—Element. anal. found (calc.) SmC_30_H_19_O_7_: %C 55.93 (56.14) and %H 2.67 (2.98); Molar conductance (Ω^−1^∙cm^2^∙mol^−1^): 5.8; IR $\bar{\nu }$ (cm^−1^): 1636 (C=O); 1594 (C=O); 1343 (C-O + O-H); 1251 (C-O-C); 560 (Sm-O). 

Complex (**2**)—Element. anal. found (calc.) EuC_30_H_23_O_9_: %C 52.83 (53.03) and %H 3.32 (3.41); Molar conductance (Ω^−1^∙cm^2^∙mol^−1^): 5.6; IR $\bar{\nu }$ (cm^−1^): 3600–2600 (broad band, O-H); 1637 (C=O); 1594 (C=O); 1581 (C=C); 1357 (C-O + O-H); 1252 (C-O-C); 562 (Eu-O). 

Complex (**3**)—Element. anal. found (calc.) GdC_30_H_21_O_8_: %C 54.18 (54.04) and %H 2.89 (3.17); Molar conductance (Ω^−1^∙cm^2^∙mol^−1^): 4.3; IR $\bar{\nu }$ (cm^−1^): 3600–2600 (broad band, O-H); 1637 (C=O); 1595 (C=O); 1582 (C=C); 1358 (C-O + O-H); 1252 (C-O-C); 562 (Gd-O). 

Complex (**4**)—Element. anal. found (calc.) TbC_30_H_21_O_8_: %C 53.63 (53.90) and %H 2.82 (3.16); Molar conductance (Ω^−1^∙cm^2^∙mol^−1^): 5.1; IR $\bar{\nu }$ (cm^−1^): 3600–2600 (broad band, O-H); 1637 (C=O); 1594 (C=O); 1581 (C=C); 1357 (C-O + O-H); 1252 (C-O-C); 563 (Tb-O).

### 3.4. Computational Study

Full geometry optimizations of the ligand and complexes were carried out using the DFT method in Gaussian09 [58]. The split valence double-zeta plus polarization basis set 6-31G(d) was applied for the carbon and hydrogen atoms. To give a better description of the lanthanide–ligand interactions, basis sets with additional diffuse functions were added for the oxygen atoms (6-31G+(d,p)). The Stuttgart-Cologne small-core quasi-relativistic pseudopotential ECP28MWB (28 electrons in the core) [59,60] was used for the Ln^3+^ ions. This ECP was used in combination with the optimized basis set: (14s13p10d8f6g)/[10s8p5d4f3g] [61]. DFT/B3LYP/(MWB28 for Ln^3+^) was proven to give good results for prediction of coordination polyhedral and geometry parameters of metal complexes in other studies [62,63].

### 3.5. DNA Binding Studies

All experiments were performed in Tris-HCl buffer [tris(hydroxymethyl)aminomethane], containing 5 mM Tris-HCl and 50 mM NaCl, adjusted to pH 7.4. The concentration of the DNA solution was determined by measuring the absorption intensity at 260 nm, using the molar extinction coefficient value of 6600 M^−1^·cm^−1^. The absorbances registered at 260 nm and 280 nm gave the ratios of ~1.8–1.9, indicating that the CT-DNA solution was sufficiently free of protein. Electronic absorption spectra were performed on a Jasco V 650 spectrophotometer using 1 cm quartz cuvettes at room temperature. Absorption titrations were determined in the range of 200–500 nm. We measured the absorbances of mixtures, containing constant concentrations of the tested complexes of 15 µM and increasing concentrations of the nucleic acid. We also measured the absorbances of mixtures, containing constant concentrations of CT-DNA (15 µM) and increasing concentrations of the tested compounds: 5, 10, 15, 20, 25, 30 µM. Each sample was kept for 10 min at room temperature to equilibrium before recording its spectrum. 

Fluorescence spectra were recorded on a Jasco FP 6500 spectrofluorometer at room temperature. A sample containing CT-DNA (10 µM) and EB (2 µM) was titrated with concentrated solutions containing the tested compounds. For every addition, the mixture was shaken and kept for 10 min at room temperature, and then the fluorescence emission spectra were recorded. The experiments have been performed in Tris-HCl/NaCl buffer (pH 7.4, 5 mM Tris-HCl/50 mM NaCl). Fluorescence emission spectra were recorded with excitation at 500 nm in the range 520–740 nm.

### 3.6. Protein Binding Studies

Fluorescence measurements were performed on a Jasco FP 6500 spectrofluorometer in a 1 cm path-length quartz cell by fluorimetric titration. A 3.0 mL sample containing 10 µM HSA was titrated by successive additions of the ligand and the complexes (ranging from 0–25 µM, with an increment of 2.5 µM). Each sample solution was shaken and kept for 3 min at the corresponding temperatures (299 K, 308 K and 318 K). The same procedure was used for Tf binding studies. Afterwards, the fluorescence spectra were recorded with the excitation wavelength set at 280 (for HSA) and 295 (for Tf) and the emission wavelength set at 290–500 nm (HSA) and 305–450 (Tf), respectively. In the meantime, the synchronous fluorescence intensities of the mixture solution (for Tf) were measured at Δλ = 60 nm and Δλ = 15 nm, respectively.

## 4. Conclusions

Primuletin (5-hydroxyflavone) forms under the selected working conditions four new complexes with Sm(III), Eu(III), Gd(III), Tb(III) ions. These complexes have been characterized by elemental analysis, thermal analysis (TG, DTA), conductometric measurements and several spectroscopic techniques (FT-IR, UV-Vis, EPR, mass spectra). From the experimental data, the composition and structure, as well as the non-electrolytic nature of the complexes have been established. In the hydroxo complexes obtained, two deprotonated molecules of ligand are bound to the metal ion in a bidentate chelate manner. The ligand and its Ln(III) complexes exhibit strong interaction with DNA, and Ln(III) complexes have much stronger DNA binding affinities than the ligand. Furthermore, the four new complexes and the ligand interacted with HSA and transferrin have been carried out and the apparent association constants (K_a_) and thermodynamic parameters (ΔH, ΔS, ΔG) have been calculated at 299 K, 308 K, and 318 K. The binding propensity varies with the binding propensity varying in the following order: (**3**) < (**1**) < (**2**) < (**4**) < 5-HOF (HSA); 5-HOF < (**3**) < (**1**)< (**4**) < (**2**) (Tf). These new lanthanide complexes show promising preliminary results, which may prove useful for the future studies. We can assume that these new complexes may be efficient tools for the in vitro study of molecular mechanisms underlying tumour development or as anticancer drugs. 

## Figures and Tables

**Table 1:** Thermal decomposition data (in air flow) for complexes (**1**)–(**4**).

Complex	Step	Thermal Effect	Temperature Range (°C)	Δ*m*_exp_ (%)	Δ*m*_calc_ (%)
(**1**)	1	Exothermic	320–1000	72.5	72.8
		Residue (Sm_2_O_3_)		27.5	27.2
(**2**)	1	Endothermic	170–300	5.0	5.3
	2	Exothermic	320–1000	72.2	72.3
		Residue (Eu_2_O_3_)		22.8	22.4
(**3**)	1	Endothermic	240–315	2.6	2.7
	2	Exothermic	325–1000	69.9	70.1
		Residue (Gd_2_O_3_)		27.5	27.2
(**4**)	1	Endothermic	210–310	2.5	2.7
	2	Exothermic	320–1000	69.6	69.9
		Residue (Tb_2_O_3_)		27.9	27.4

**Table 2:** Geometric parameters bond lengths (A), bond angles (°), charge density, total energy (a.u.) and total dipole moment (D) of 5-hydroxyflavone as resulted from DFT calculations.

**Bond Length (A)**			
C4-O7	1.351		
C9=O27	1.221		
**Bond Angle (°)**			
C4-C5-C9	122.998		
O7-C4-C5	120.781		
O27-C9-C5	121.151		
**Charge**			
C4	0.836	O7	−0.434
C9	0.448	O27	−0.469
**Total energy (a.u.)**		−802.4868	
**Total dipole moment (D)**		4.3673	

**Table 3:** Geometric parameters bond lengths (A), angles (°), charge density, total energy and total dipole moment of complexes (**1**)–(**4**) as resulted from DFT calculations.

Parameter	Complex			
	(1)	(2)	(3)	(4)
**Bond Length (A)**				
M-O7	2.155	2.149	2.156	2.150
M-O27	2.132	2.120	2.233	2.201
M-O29	2.167	2.151	2.252	2.259
M-O30	2.149	2.146	2.177	2.124
M-O31	2.361	2.485	2.395	2.395
M-O58	-	2.410	2.379	2.378
C9-O27	1.276	1.304	1.306	1.307
C4-O7	1.281	1.312	1.310	1.315
**Bond Angle (°)**				
O27-M-O31	54.577	48.731	51.462	51.624
O27-M-O7	73.051	71.572	71.167	71.512
O7-M-O29	111.091	105.489	97.734	97.707
O30-M-O31	46.376	49.686	53.136	50.077
O30-M-O29	74.919	71.119	69.736	72.046
O29-M-O58	-	57.411	48.829	49.279
O58-M-O7	-	50.274	51.109	50.623
C4-C5-C9	122.998	120.776	119.586	121.189
O7-C4-C5	120.781	120.263	121.871	120.425
**Dihedral Angle**				
M-O27-C9-C5	−1.366	−21.069	−30.917	−30.948
M-O7-C4-C5	−3.011	18.903	18.397	18.769
O31-M-O7-C4	−1.855	−41.144	−47.061	−47.380
M-O7-C4-C3	179.599	−158.486	−156.593	−156.435
M-O29-C33-C37	164.413	136.736	139.683	145.150
**Charge**				
M	1.079	1.186	0.887	1.076
O7	−0.705	−0.585	−0.541	−0.556
O27	−0.611	−0.377	−0.395	−0.344
O29	−0.639	−0.468	−0.383	−0.396
O30	−0.568	−0.529	−0.592	−0.556
O31	−0.501	−0.521	−0.416	−0.549
O58	−	−0.635	−0.626	−0.628
C4	0.406	0.228	0.221	0.213
C9	0.561	0.206	0.163	0.121
C33	0.554	0.255	0.141	0.162
C38	0.406	0.221	0.281	0.233
**Total energy (a.u.)**	−1706.6004	−2467.6373	−2522.8014	−2581.6789
**Total dipole moment (D)**	4.3164	7.2773	6.7800	5.6414

**Table 4:** Binding constants for 5-HOF and complexes (**2**), (**3**), (**4**), respectively and CT-DNA interaction systems.

Compound	K_b_ (L∙mol^−1^)	R^2^
**5-HOF**	1.47 × 10^4^	0.9999
(**2**)	2.73 × 10^4^	0.994
(**3**)	2.93 × 10^4^	0.9934
(**4**)	3.85 × 10^4^	0.9981

**Table 5:** Quenching constants of the interaction between the tested compounds and CT-DNA-EB complex.

Compound	K_sv_ (L∙mol^−1^)	R^2^
**5-HOF**	2.89 × 10^4^	0.9928
(**2**)	1.73 × 10^5^	0.9959
(**3**)	4.13 × 10^5^	0.9941
(**4**)	1.39 × 10^5^	0.9902

## Supplementary Materials

Supplementary materials can be accessed at: http://www.mdpi.com/1420-3049/21/12/1737/s1.
