# Supplementary material for: Synthesis and Structural Investigation of New Bio-Relevant Complexes of Lanthanides with 5-Hydroxyflavone: DNA Binding and Protein Interaction Studies
Source: Molecules. 2016 Dec 16;21(12):1737. doi: 10.3390/molecules21121737 (PMC6273368; doi:10.3390/molecules21121737)
Supplement: Supplementary file 1 [file molecules-21-01737-s001.pdf]

Supplementary Materials: Synthesis and Structural Investigation of New Bio-Relevant Complexes of Lanthanides with 5-Hydroxyflavone: DNA Binding and Protein Interaction Studies

Alexandra-Cristina Munteanu, Mihaela Badea, Rodica Olar, Luigi Silvestro, Constanţa Dulea, Constantin-Daniel Negut and Valentina Uivarosi

Table S1. UV-Vis data for ligand and complexes.

Compound	λ_{max} (nm)		
	Band I		Band II
**5-HOF**	385	340	255
(**1**)	405	320	265
(**2**)	395	315	270
(**3**)	395	315	270
(**4**)	395	320	270

Table S2. Nominal *m*/*z* of the ions observed in the positive ion mode spectra of the complexes—Colums 2, 3, 4 pseudomolecular ions by ESI-MS positive, column 5 main MS/MS fragments from the protonated pseudomolecular ions (see *m*/*z* in column 2).

Compound	*m*/*z* Value Fragment Ion	*m*/*z* Value Fragment Ion	*m*/*z* Value Fragment Ion	*m*/*z* Value Fragment Ion
(**1**)	-	626	644	662
		$^{152}SmL_2^+$	$^{152}SmL_2(H_2O)^+$	$^{152}SmL_2(H_2O)_2^+$
(**2**)	406	625	643	661
	$[^{151}Eu(OH)L]^+$	$^{151}EuL_2^+$	$^{151}EuL_2(H_2O)^+$	$^{151}EuL_2(H_2O)_2^+$
	408	627	645	663
	$[^{153}Eu(OH)L]^+$	$^{153}EuL_2^+$	$^{153}EuL_2(H_2O)^+$	$^{153}EuL_2(H_2O)_2^+$
(**3**)	414	634	652	670
	$[^{160}Gd(OH)L]^+$	$^{160}GdL_2^+$	$^{160}GdL_2(H_2O)^+$	$^{160}GdL_2(H_2O)_2^+$
(**4**)	413	633	651	669
	$[^{159}Tb(OH)L]^+$	$^{159}TbL_2^+$	$^{159}TbL_2(H_2O)^+$	$^{159}TbL_2(H_2O)_2^+$

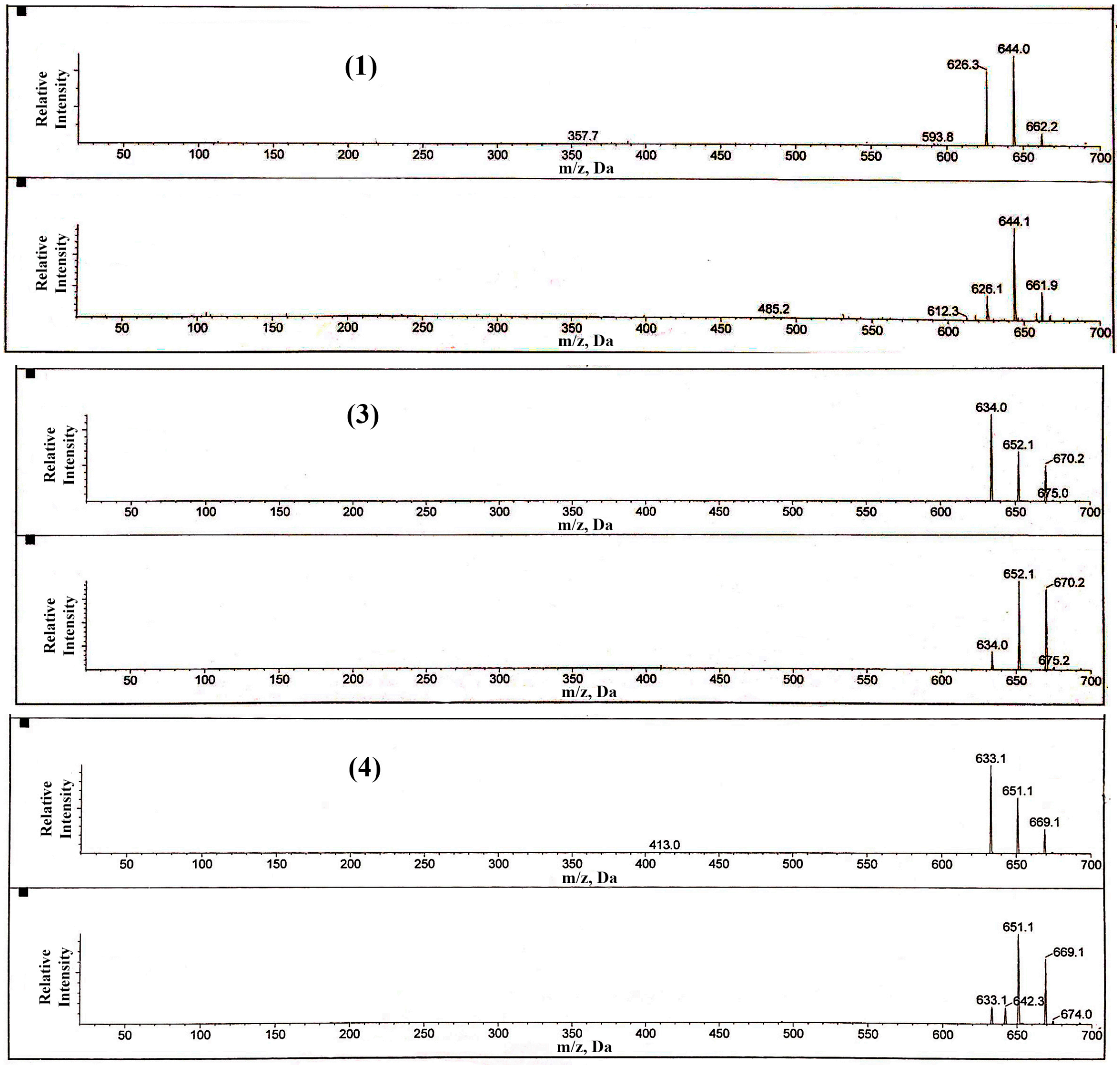

(**A**)

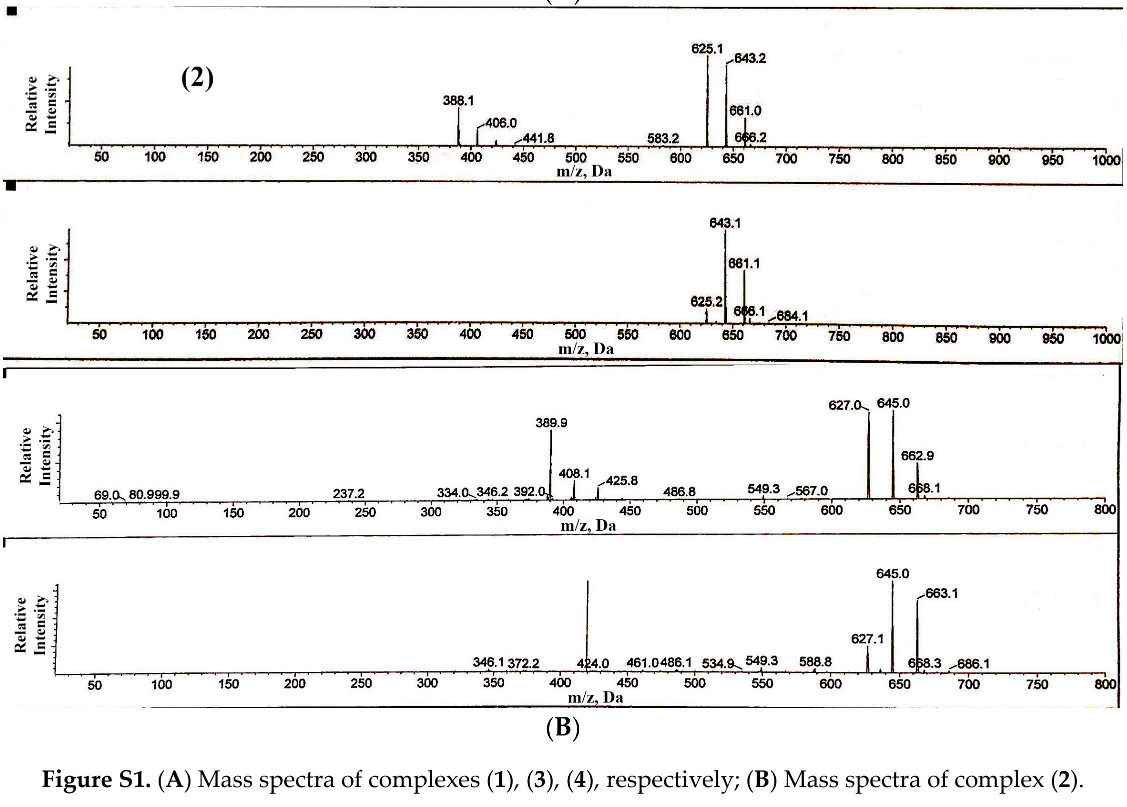

(**B**)

Figure S1. (**A**) Mass spectra of complexes (**1**), (**3**), (**4**), respectively; (**B**) Mass spectra of complex (**2**).

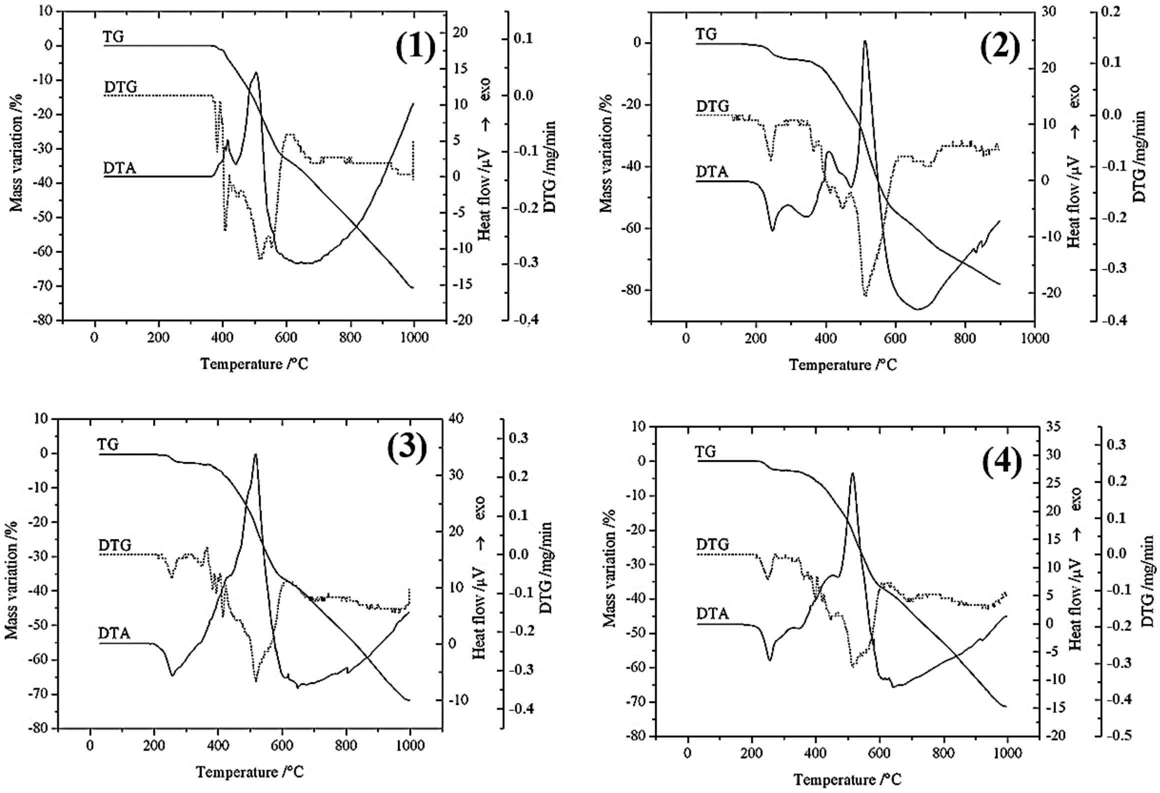

Figure S2. Thermogravimetric analysis of complexes (**1**)–(**4**), respectively.

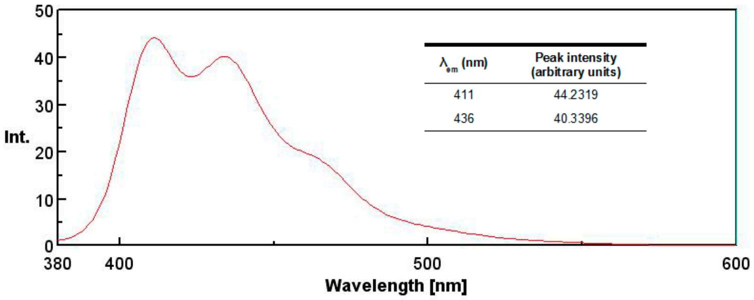

Figure S3. Fluorescence spectra of **2** in chloroform solution; λ_{exc} = 360 nm.

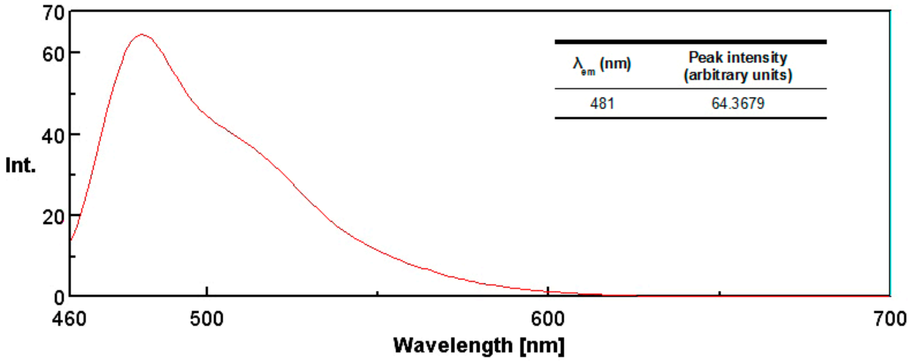

Figure S4. Fluorescence spectra of (**1**) in chloroform solution; λ_{exc} = 440 nm.

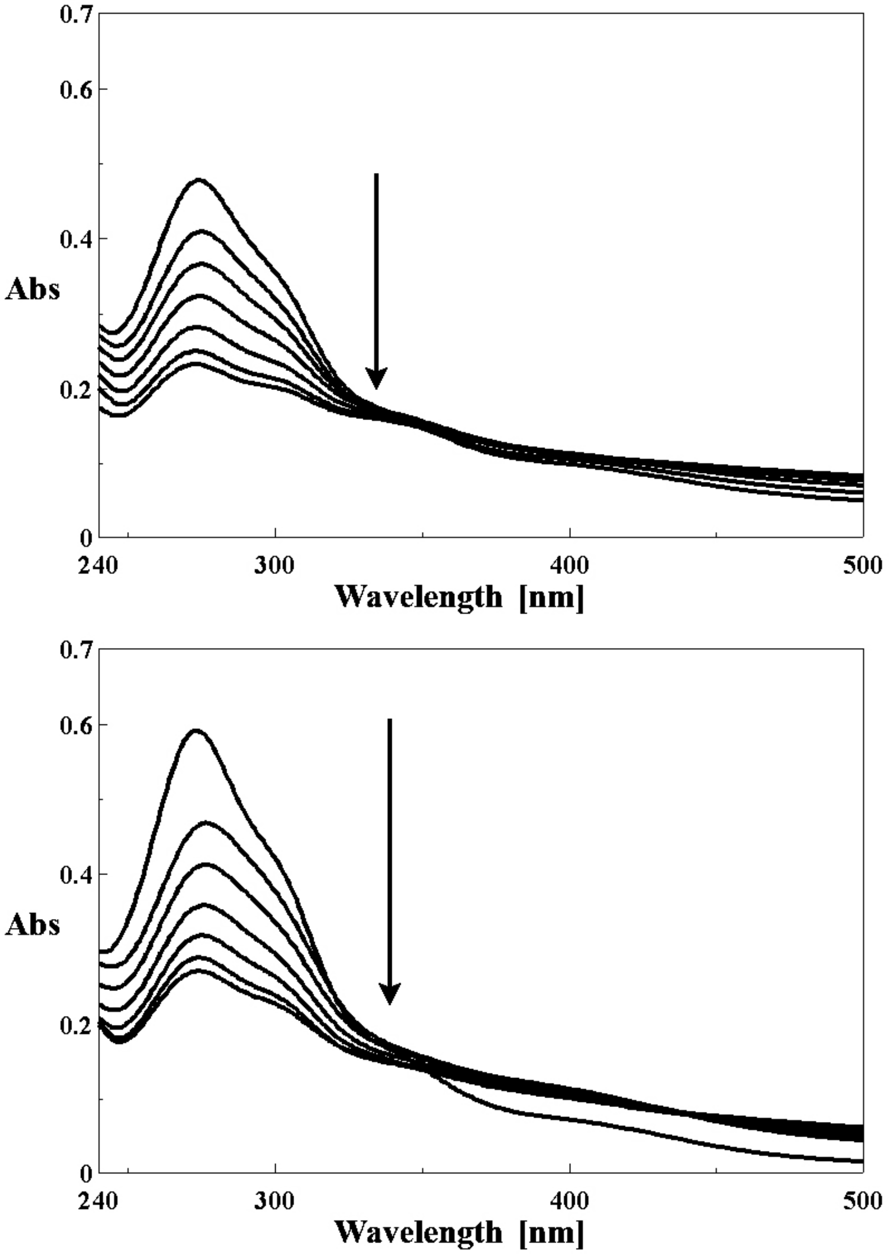

Figure S5. Absorption spectra of complexes (**3**), (**4**) in the absence and presence of increasing amounts of DNA. [complex] = 15 μM; [DNA] = 0; 5; 10; 15; 20; 25; 30 μM. The arrows show the absorption changes on increasing DNA concentration.

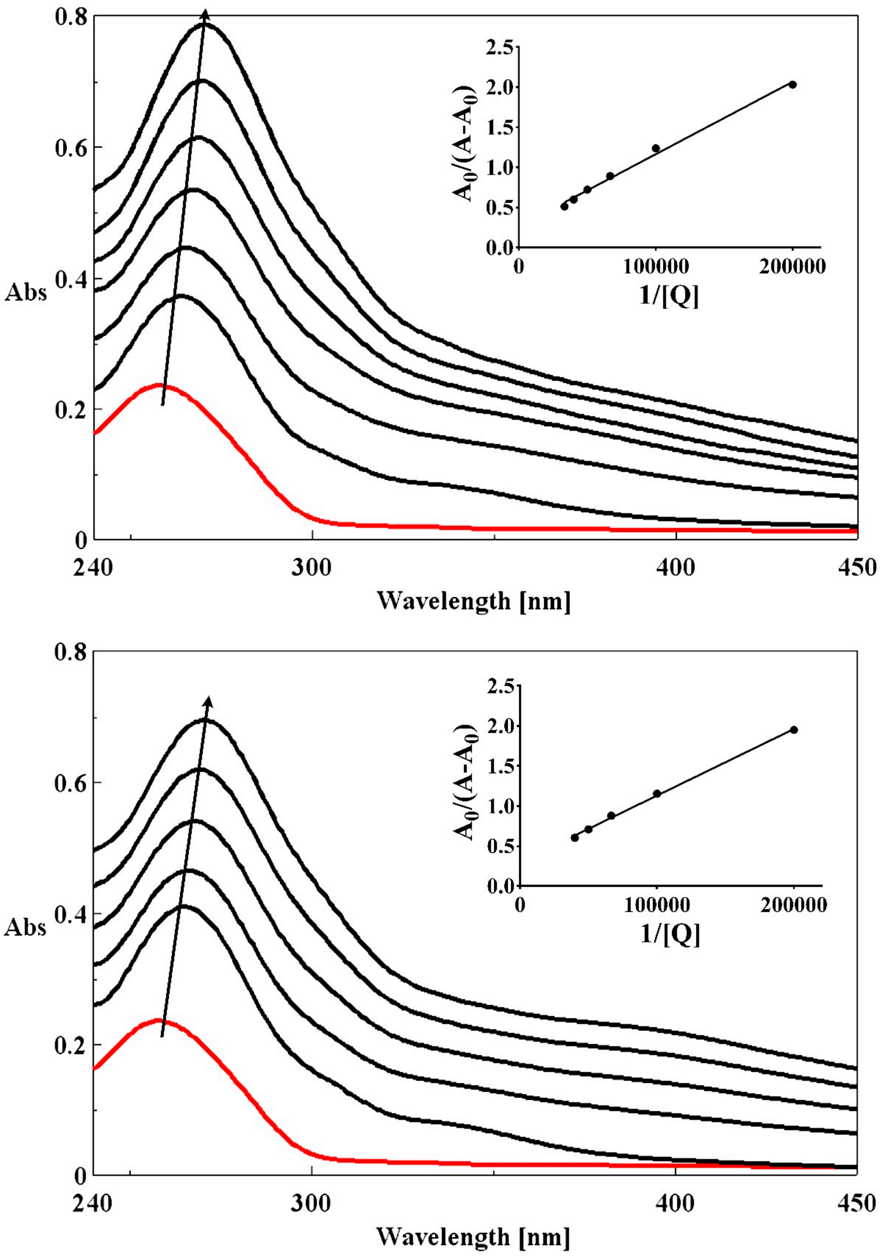

Figure S6. Absorption spectra of DNA in the absence and presence of increasing amounts of complexes (**3**)—first spectrum, (**4**)—second spectrum. [DNA] = 15 μM; [compound] = 0; 5; 10; 15; 20; 25; 30 μM. Insert figures represent the plots of $A_0/(A - A_0)$ vs. 1/[Q]. The arrows show the absorption changes on increasing concentration of the tested compound.

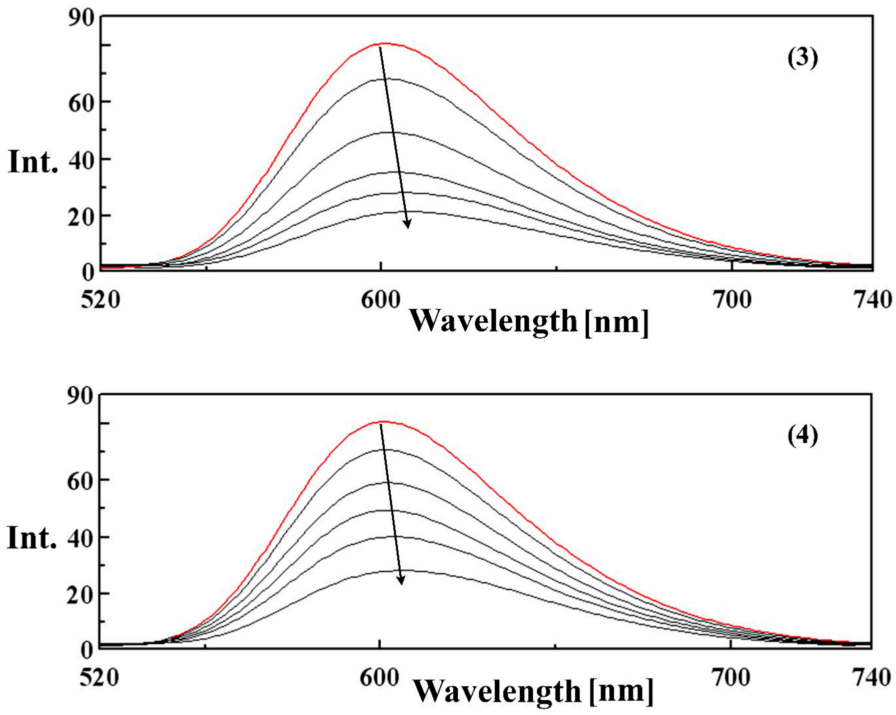

Figure S7. Fluorescence spectra of the binding of ethidium bromide (EB) to DNA in the absence (red line) and in the presence (black lines) of increasing amounts of the ligand and complexes (**3**) and (**4**), respectively. λ_{ex} = 500 nm, [EB] = 2 μM, [DNA] = 10 μM, [compound] = 10, 15, 20, 25, 30 μM. Arrows indicate the changes in fluorescence intensities upon increasing the amounts of the tested compound.

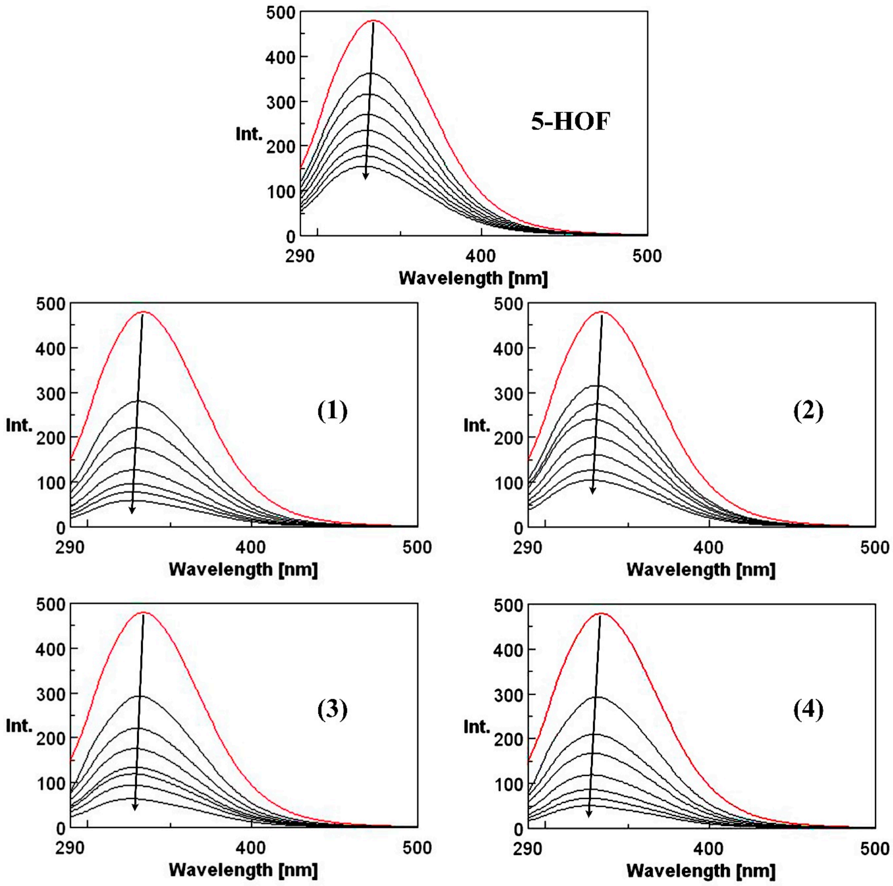

Figure S8. Fluorescence spectra of 5-HOF and complexes (**1**)–(**4**)—HSA systems at 299 K.

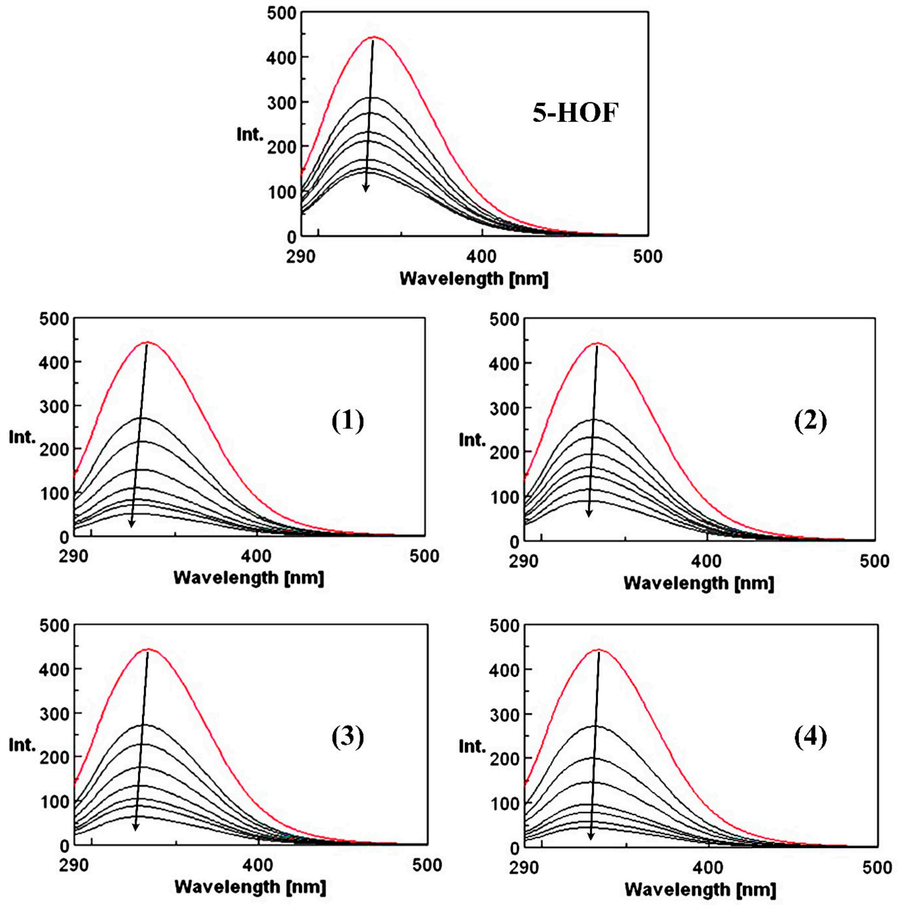

Figure S9. Fluorescence spectra of 5-HOF and complexes (**1**)–(**4**)—HSA systems at 308 K.

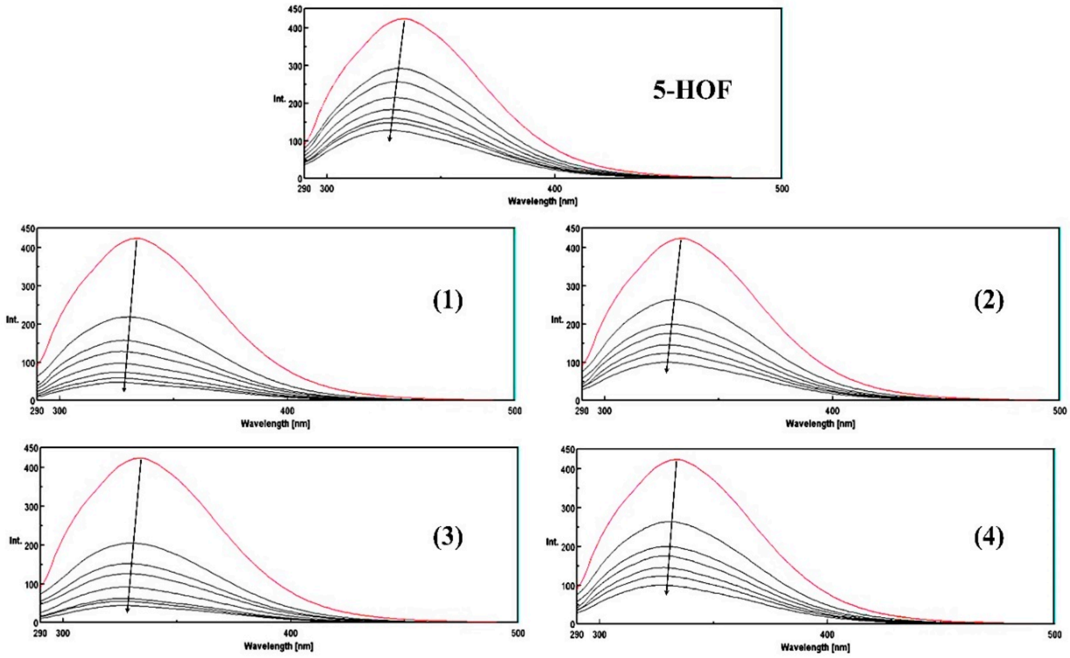

Figure S10. Fluorescence spectra of 5-HOF and complexes (**1**)–(**4**)—HSA systems at 318 K.

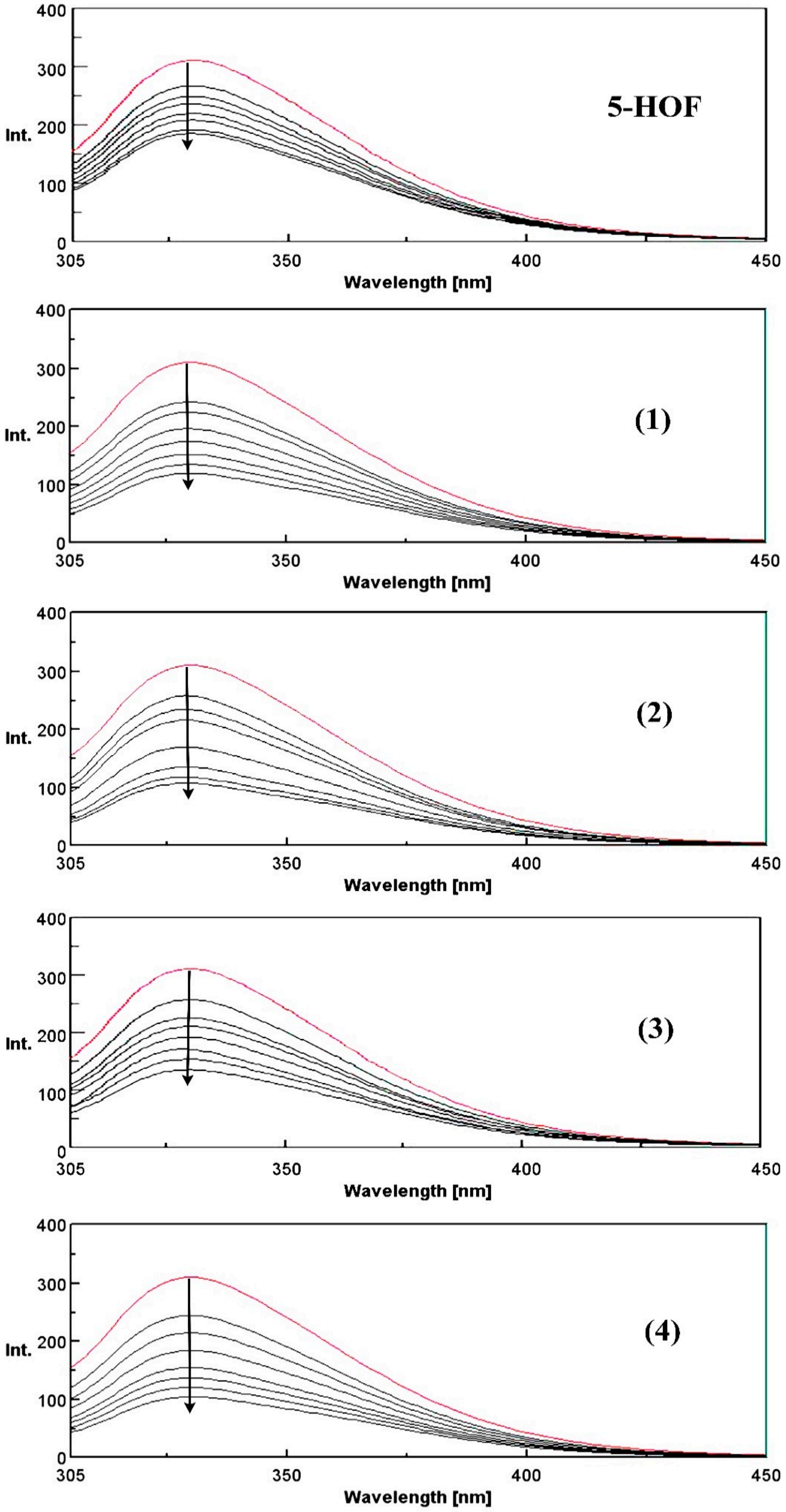

Figure S11. Fluorescence spectra of 5-HOF and complexes (**1**)–(**4**)—Tf systems at 299 K.

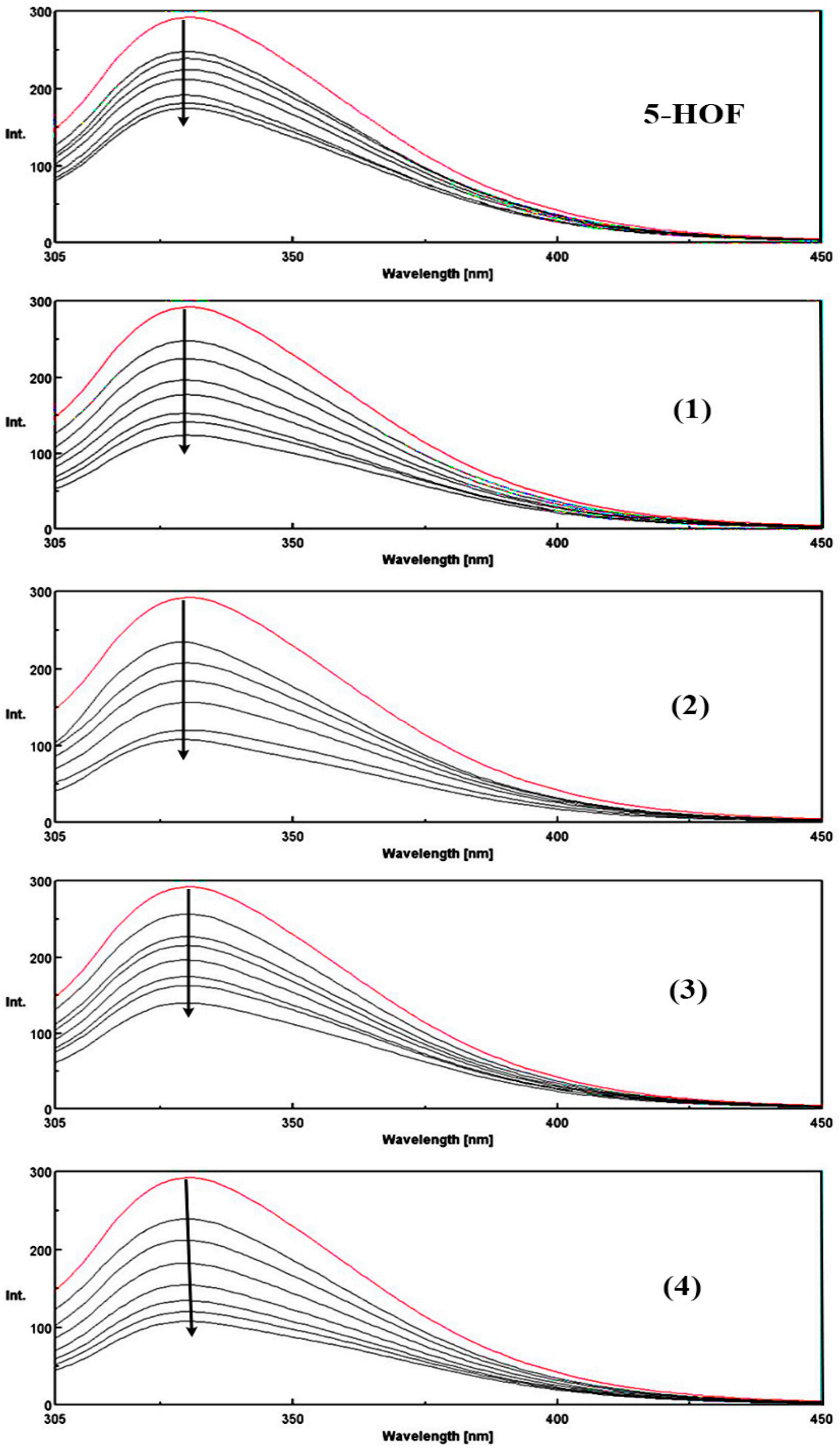

Figure S12. Fluorescence spectra of 5-HOF and complexes (**1**)–(**4**)—Tf systems at 308 K.

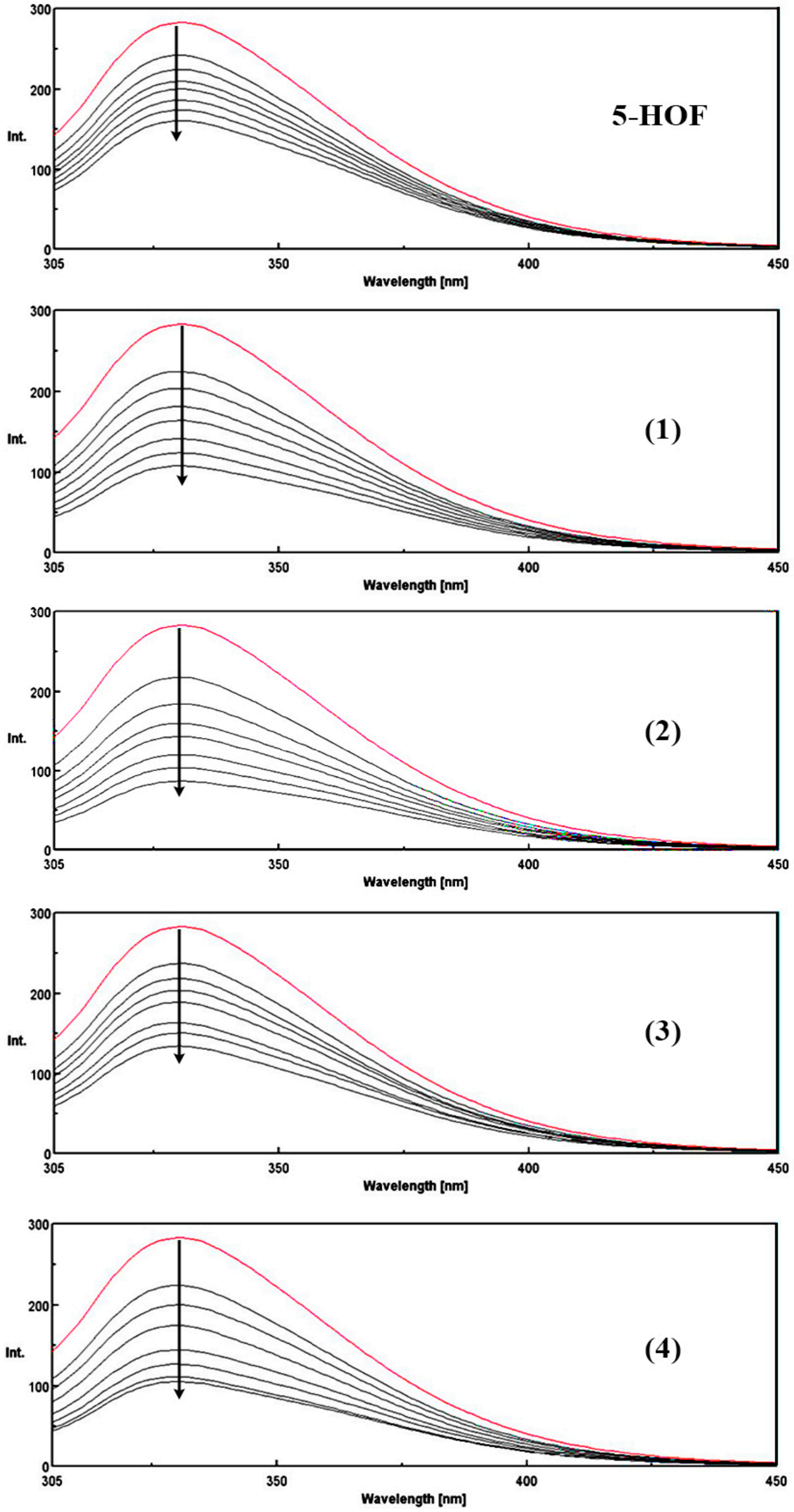

Figure S13. Fluorescence spectra of 5-HOF and complexes (**1**)–(**4**)—Tf systems at 318 K.

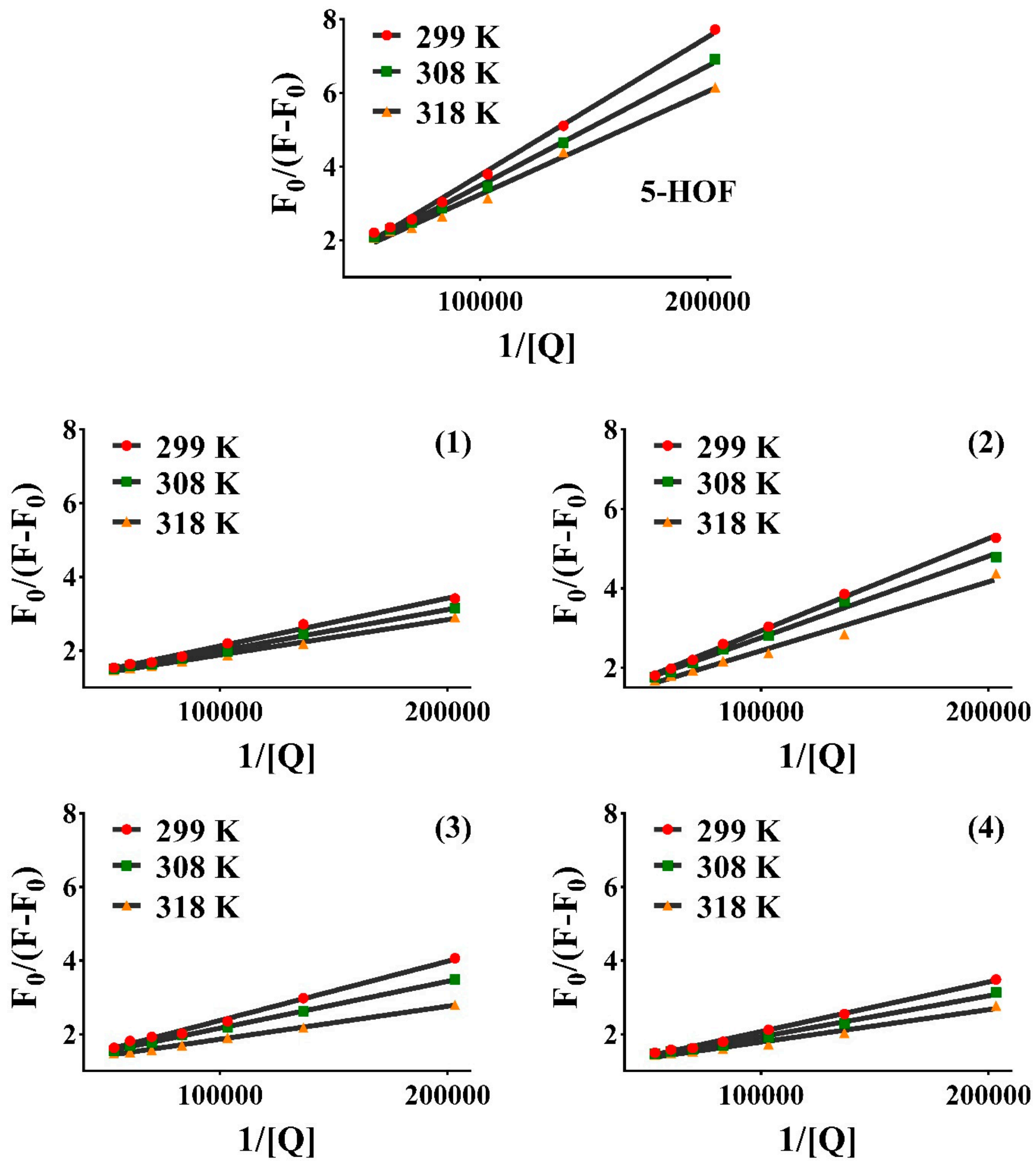

Figure S14. Modified Stern-Volmer plots of 5-HOF, (**1**)—(**4**)—HSA systems at three different temperatures.

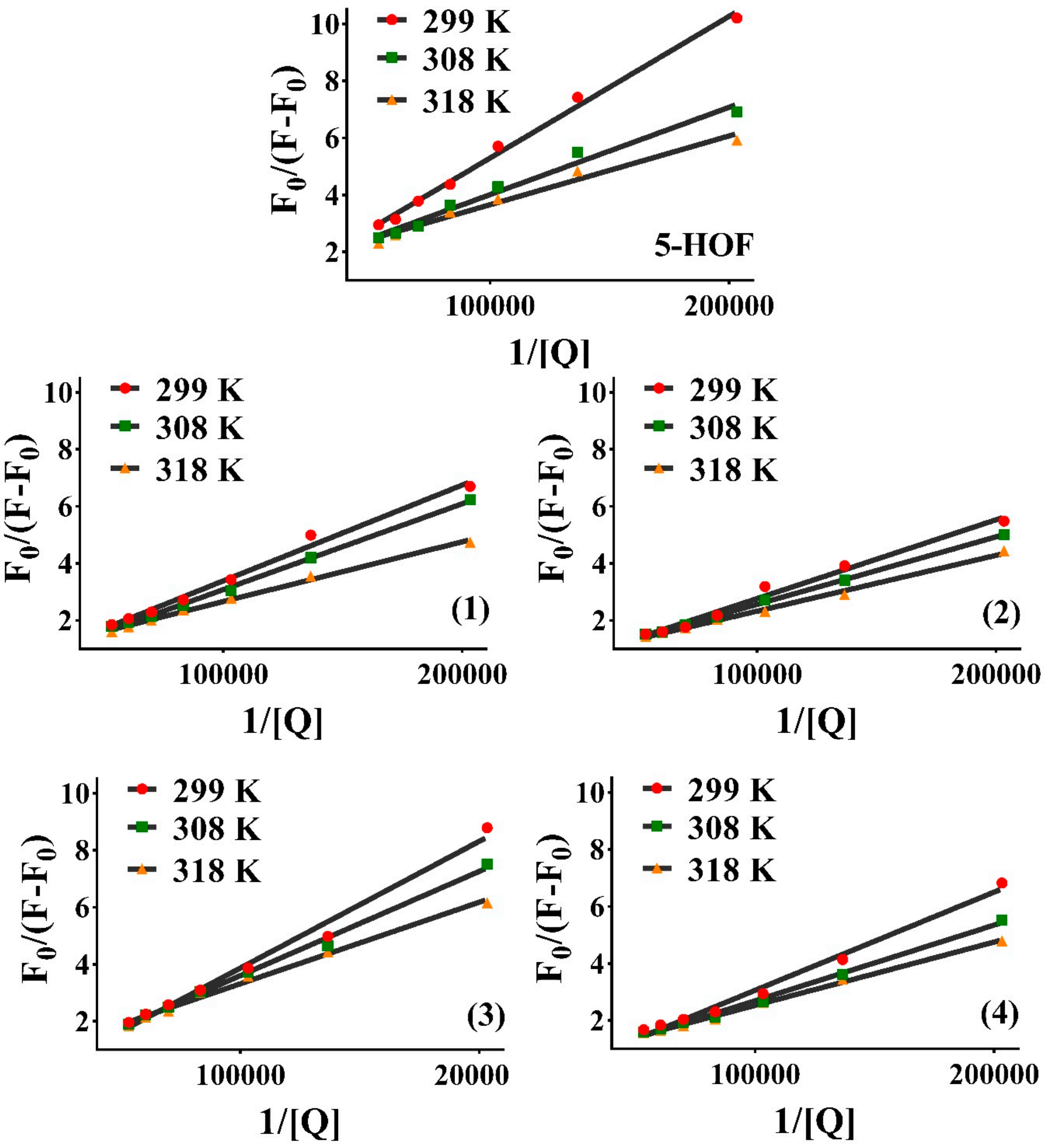

Figure S15. Modified Stern-Volmer plots of 5-HOF, (**1**)–(**4**)—Tf systems at three different temperatures.

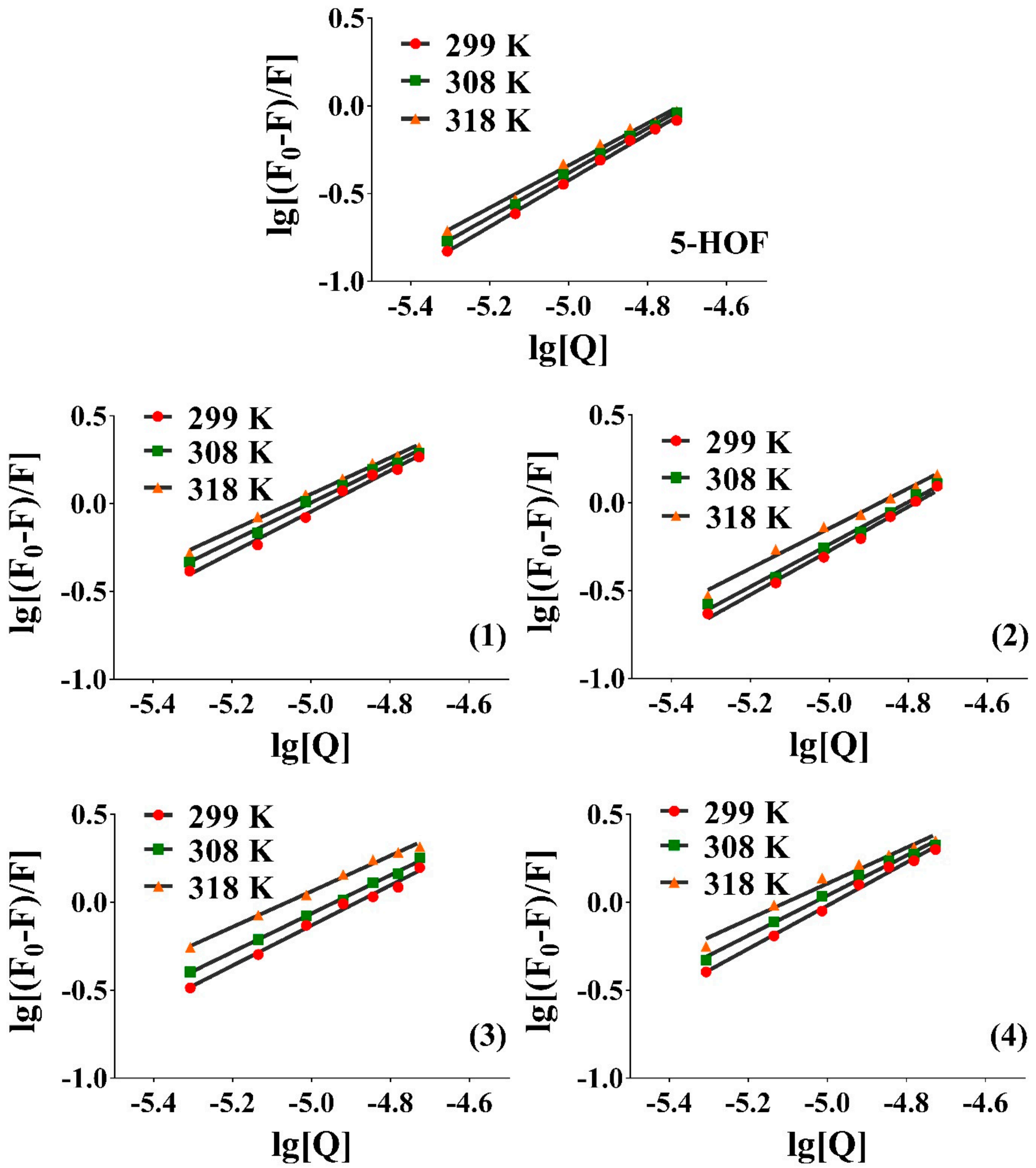

Figure S16. Plots of lg[(F_0 − F)/F] vs. lg[Q] for the HSA- tested compounds systems (5-HOF, (**1**)–(**4**), respectively) at three different temperatures; [HSA] = 10 μM, [compound] = 5; 7.5; 10; 12.5; 15; 17.5; 20 μM.

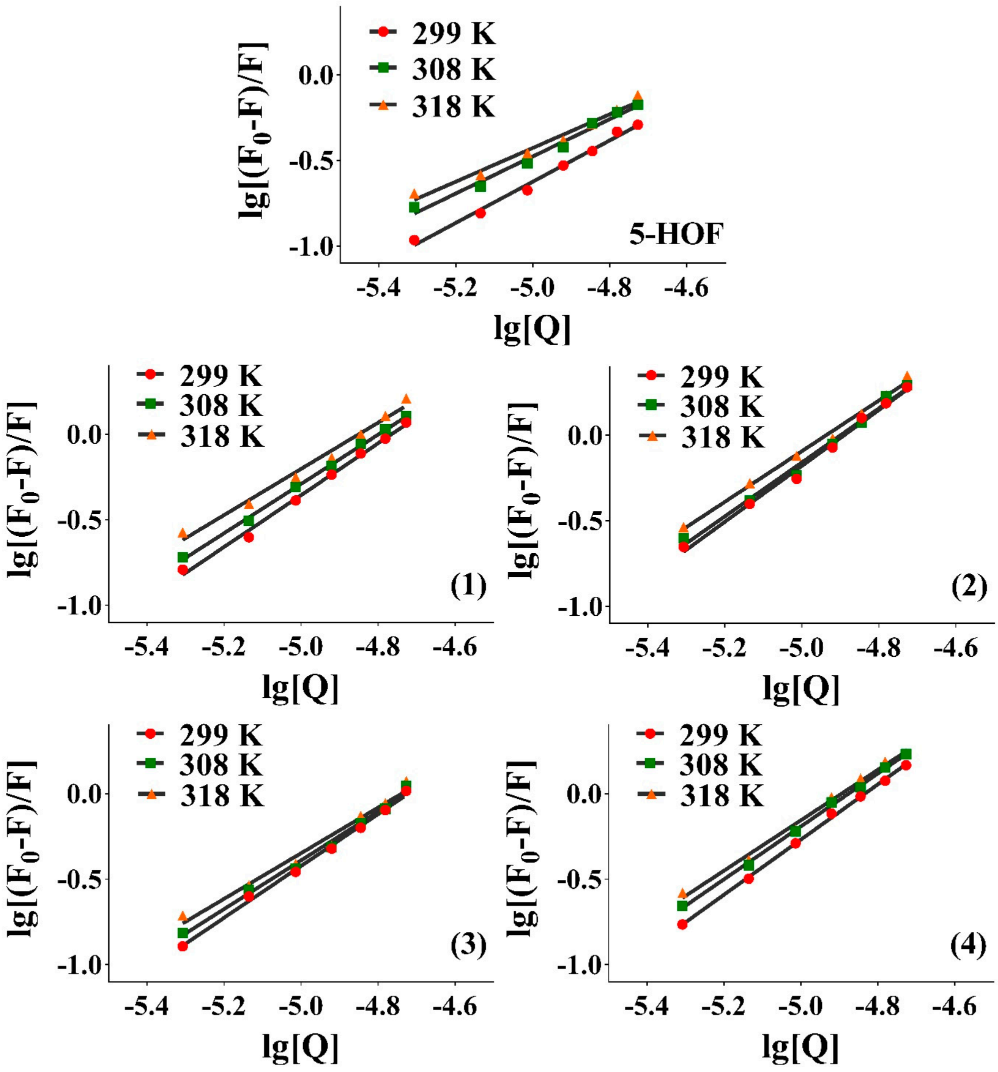

Figure 17. Plots of lg[(F_0 − F)/F] vs. lg[Q] for the 5-HOF and (**1**)–(**4**)—Tf systems, respectively, at three different temperatures; [Tf] = 10 μM, [compound] = 5; 7.5; 10; 12.5; 15; 17.5; 20 μM.

Table S3. The binding constants and thermodynamic parameters for HSA-tested compounds interaction.

Compound	T (K)	Ksv (M^{-1})	Kq ($M^{-1}\cdot s^{-1}$)	Ka (M^{-1})	*n*	ΔH ($kJ\cdot mol^{-1}$)	ΔS ($J\cdot mol^{-1}\cdot K^{-1}$)	ΔG ($kJ\cdot mol^{-1}$)
	299	4.64×10^{3}	4.64×10^{11}	1.51×10^{6}	1.32			−35.42
**5-HOF**	308	8.30×10^{3}	8.30×10^{11}	9.18×10^{5}	1.27	−47.51	−40.44	−35.05
	318	1.50×10^{4}	1.50×10^{12}	4.84×10^{5}	1.20			−34.65
	299	8.32×10^{4}	8.32×10^{12}	5.93×10^{5}	1.16			−32.99
(**1**)	208	8.82×10^{4}	8.82×10^{12}	3.01×10^{5}	1.09	−51.81	−62.94	−32.42
	318	9.31×10^{4}	9.31×10^{12}	1.70×10^{5}	1.04			−31.80
	299	3.02×10^{4}	3.02×10^{12}	9.75×10^{5}	1.25			−34.33
(**2**)	308	3.58×10^{4}	3.58×10^{12}	6.29×10^{5}	1.21	−41.62	−24.39	−34.11
	318	3.96×10^{4}	3.93×10^{12}	3.59×10^{5}	0.14			−33.86
	299	3.84×10^{4}	3.84×10^{12}	3.82×10^{5}	1.14			−32.03
(**3**)	308	9.05×10^{4}	9.05×10^{12}	2.53×10^{5}	1.09	−42.80	−36.02	−31.71
	318	1.08×10^{5}	1.08×10^{13}	1.37×10^{5}	1.01			−31.35
	299	7.29×10^{4}	7.29×10^{12}	1.29×10^{6}	1.23			−35.08
(**4**)	308	8.19×10^{4}	8.19×10^{12}	5.21×10^{5}	1.14	−87.29	−174.60	−33.51
	318	1.01×10^{5}	1.01×10^{13}	1.59×10^{5}	1.02			-31.77

Table S4. The binding constants and thermodynamic parameters for Tf-tested compounds interaction.

Compound	T (K)	Ksv (M^{-1})	Kq ($M^{-1}{\cdot}s^{-1}$)	Ka (M^{-1})	*n*	ΔH ($kJ{\cdot}mol^{-1}$)	ΔS ($J{\cdot}mol^{-1}{\cdot}K^{-1}$)	ΔG ($kJ{\cdot}mol^{-1}$)
**5-HOF**	299	6.57×10^3	2.63×10^{12}	2.53×10^5	1.21	−86.36	−185.46	−30.89
	308	3.16×10^4	1.26×10^{13}	8.91×10^4	1.09			−30.29
	318	6.22×10^4	2.49×10^{13}	3.17×10^4	0.99			−29.62
(**1**)	299	8.93×10^2	3.57×10^{11}	1.65×10^7	1.51	−64.16	−76.29	−41.35
	308	5.43×10^3	2.17×10^{12}	8.16×10^6	1.44			−40.66
	318	2.8×10^4	1.12×10^{13}	3.53×10^6	1.35			−39.90
(**2**)	299	4.76×10^3	1.90×10^{12}	9.73×10^7	1.63	−53.12	−24.62	−45.76
	308	7.49×10^3	2.99×10^{12}	5.41×10^7	1.58			−45.54
	318	1.84×10^4	7.36×10^{12}	2.71×10^7	1.51			−45.29
(**3**)	299	1.08×10^3	3.6×10^{11}	1.42×10^7	1.52	−79.45	−128.91	−40.91
	308	7.15×10^3	2.86×10^{12}	5.38×10^6	1.42			−39.75
	318	1.51×10^4	6.04×10^{12}	2.10×10^6	1.33			−38.46
(**4**)	299	1.1×10^2	4.4×10^{10}	6.30×10^7	1.61	−55.53	−36.26	−44.68
	308	1.35×10^3	5.4×10^{11}	3.47×10^7	1.55			−44.36
	318	1.56×10^4	6.25×10^{12}	1.66×10^7	1.47			−43.99

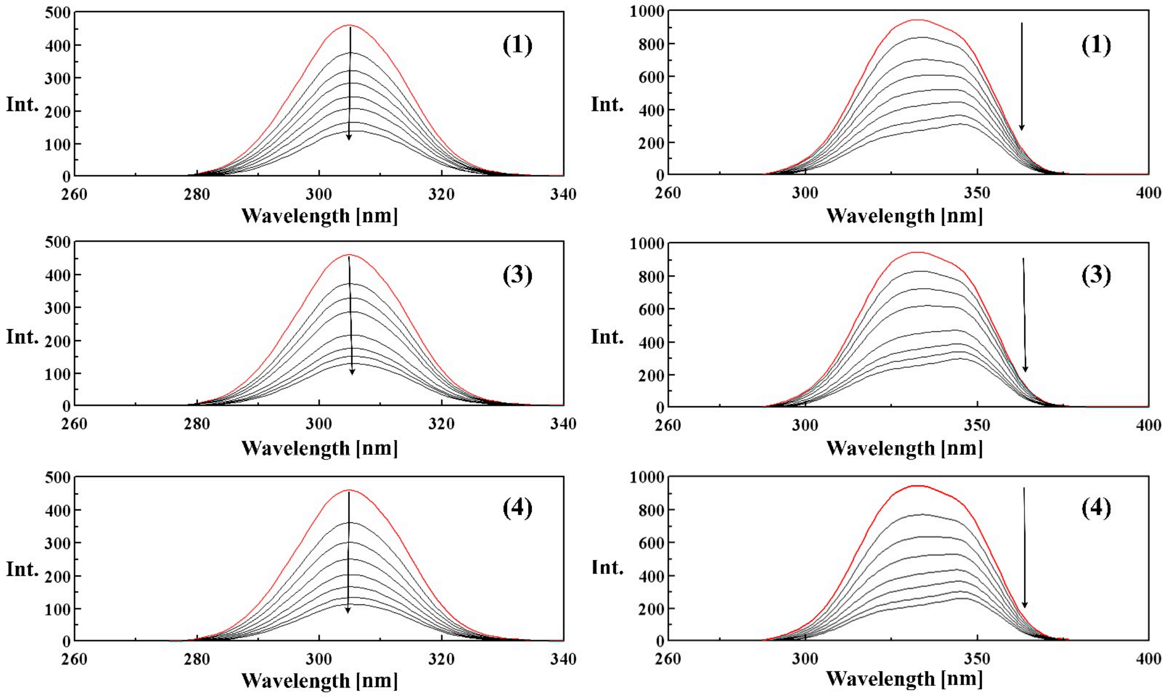

Figure S18. Fluorescence synchronous spectra of 5-HOF and complexes (**1**), (**3**), (**4**), respectively at Δλ = 15 nm (**left**) and 60 nm (**right**). [Tf] = 10 μM, pH = 7.4; [tested compound] = 0; 5; 7.5; 10; 12.5; 15; 17.5; 20 μM. Arrows indicate the changes in fluorescence intensities upon increasing the amounts of the tested compound.
